# Quantitative Resistance Deployment Can Strengthen Epidemics in Perennial Plants by Selecting Maladapted Pathogen Strains

**DOI:** 10.1111/eva.70123

**Published:** 2025-07-09

**Authors:** Jean‐Paul Soularue, Fabien Halkett, Méline Saubin, Sukanya Denni, Arthur Demené, Cyril Dutech, Cécile Robin

**Affiliations:** ^1^ Univ. Bordeaux, INRAE, BIOGECO Pessac France; ^2^ Université de Lorraine, INRAE, IAM Nancy France

**Keywords:** evolutionary epidemiology, host–microbe interaction, nested modeling, plant pathogen, resistance deployment strategy, virulence evolution

## Abstract

Quantitative resistances are essential tools for mitigating epidemics in managed plant ecosystems. However, their deployment can drive evolutionary changes in pathogen life‐history traits, making predictions of epidemic development challenging. To investigate these effects, we developed a demo‐genetic model that explicitly captures feedbacks between the pathogen's population demography and its genetic composition. The model also links within‐host multiplication and between‐host transmission, and is built on the assumption that the coexistence of susceptible and resistant hosts imposes divergent selection pressures on the pathogen population at the landscape scale. We simulated contrasting landscapes of perennial host plants with varying proportions of resistant plants and resistance efficiencies. Our simulations confirmed that deploying resistances with nearly complete efficiency (> 99.99%) effectively reduces the severity of epidemics caused by pathogen introduction and promotes the specialization of infectious genotypes to either susceptible or resistant hosts. Conversely, the use of partial resistances induces limited evolutionary changes, often resulting in pathogen maladaptation to both susceptible and resistant hosts. Notably, deploying resistances with strong (89%) or moderate (60%) efficiencies can, under certain conditions, lead to higher host mortality compared to entirely susceptible populations. This counterintuitive outcome arises from the maladaptation of infectious genotypes to their hosts, which prolongs the lifespan of infected hosts and can increase inoculum pressure. We further compared simulations of the full model with those of simplified versions in which (i) the contribution of infected plants to disease transmission did not depend on the pathogen load they carried, (ii) plant landscapes were not spatially explicit. These comparisons highlighted the essential role of these components in shaping model predictions. Finally, we discuss the conditions that may lead to detrimental outcomes of quantitative resistance deployments in managed perennial plants.

## 1 Introduction

The management of emerging plant diseases often involves using selected plants that inherently resist the pathogens. This approach can become ineffective due to the rapid adaptation of pathogens to the resistances being used (Thrall and Burdon 2003; Rex Consortium 2016). One major challenge is accurately predicting how newly introduced plant pathogens will evolve and propagate in the presence of a diversity of host resistances (Gilligan 2008; Rimbaud et al. 2021). To effectively address this challenge, there is a need for models that incorporate the biological characteristics of the pathosystems in question (Lannou 2012; Saubin et al. 2023, 2024).

Existing models of plant resistance deployment typically describe simple heterogeneous landscapes composed of two classes of hosts, resistant and susceptible, exposed to an expanding population of pathogens (but see also Sapoukhina et al. 2013; Mikaberidze et al. 2015; Clin et al. 2022). Many of these models are built on the assumption that a pathogen's adaptation to one type of plant (initially resistant or susceptible) results in its reduced fitness on the other type (e.g., Leach et al. 2001; Pietravalle et al. 2006; Montarry et al. 2012; Djidjou‐Demasse et al. 2017; Rimbaud et al. 2021). When the models incorporate pathogen evolution, they provide predictions about both epidemic development and genetic changes within pathogen populations, which often occur simultaneously (Day and Proulx 2004). Interestingly, the relationship between the proportion of disease‐resistant plants in a population and the overall incidence of the disease is not straightforward and may be non‐monotonic (Papaïx et al. 2018). This relationship may vary depending on several factors, such as the efficiency of the resistance used (Iacono et al. 2012; Ellis et al. 2014; Rimbaud et al. 2023), the degree of variability in plant defense mechanisms (van den Bosch and Gilligan 2003; Laine and Tellier 2008; Fabre et al. 2022), the life cycle of the pathogen (Xu 2012; Saubin et al. 2021), or the time scale considered (van den Bosch and Gilligan 2003; Papaïx et al. 2018; Taylor and Cunniffe 2023). Moreover, the speed at which pathogens adapt to the resistance is also non‐monotonically affected by the proportion of resistant hosts in the population (Fabre et al. 2022). Therefore, determining the optimal proportion of resistant plants to minimize both pathogen adaptation and disease spread remains a complex and highly context‐dependent task.

Quantitative resistances provide graded levels of protection to plants against pathogens. Often considered more durable than qualitative resistances which offer all‐or‐nothing protections (Parlevliet 2002; Chen et al. 2003; Schnurbusch et al. 2004; Palloix et al. 2009; Langlands‐Perry et al. 2023), they are increasingly recognized as valuable tools for protecting plant populations against disease outbreaks (Niks et al. 2015). The outcome of quantitative host–pathogen interactions may depend on the expression of quantitative traits under polygenic determinism in both the plant and the pathogen (Corwin and Kliebenstein 2017; Andersen et al. 2018). In such a scenario, the selection of beneficial allelic associations produces additive covariances that influence the phenotypic variance maintained in expanding pathogen populations and ultimately shape their adaptive trajectories (Whitlock and Guillaume 2009; Le Corre and Kremer 2012; Walsh and Lynch 2018). We advocate that the quantitative genetic framework is an opportunity to build models that comprehensively incorporate the genetic architecture and heritability of the pathogen's quantitative traits and the selective pressures arising from the coexistence of susceptible and resistant hosts (Day and Proulx 2004; Walsh and Lynch 2018). In particular, this approach enables the transposition of the largely considered gene‐for‐gene (Flor 1971) model and adaptation costs concepts (e.g., Thrall and Burdon 2003; Bahri et al. 2009; Laine and Barrès 2013) into a quantitative and polygenic context. This can be achieved by considering divergent selection of varying intensity on the adaptive trait of interest, as recently done by Fabre et al. (2022).

A common feature of existing models is that pathogen transmission from infected to healthy hosts is often assumed to be fixed or phenomenologically modulated by the pathogen's ability to infect and colonize hosts. In some studies, transmission‐related components such as the number of infectious spores in fungi and the latency period, are specifically targeted by deployed resistance and can evolve (e.g., van den Berg et al. 2014; Fabre et al. 2022). However, the intricate process by which pathogens colonize infected plants is rarely linked mechanistically to the broader dynamics of disease transmission between plants. This decoupling specifically prevents the mechanistic emergence of variations in disease transmission among plants, that could reflect virulence‐transmission trade‐offs (Alizon et al. 2009) not defined a priori. Consequently, this limitation may restrict the insights provided by such models (Gilchrist and Coombs 2006; Mideo et al. 2008; Elie et al. 2022; Jiranek et al. 2023), particularly for perennial plants, which are progressively colonized by pathogens over time and can contribute to disease spread for many years.

We investigated the outcomes of deploying quantitative resistance in managed populations of perennial plants at risk of disease emergence. To achieve this, we developed and explored a novel agent‐based model to study the evolution and epidemiology of an introduced pathogenic fungus. This fungus reproduces sexually and causes new infections via airborne spores. The model's development was driven by the need to address severe tree epidemics in both temperate and tropical regions (Santini et al. 2013; Desprez‐Loustau et al. 2016). Notable examples include ash dieback in Europe caused by *Hymenoscyphus fraxineus* (Landolt et al. 2016), Sphaeropsis blight caused by *Diplodia sapinea* (Decourcelle et al. 2015), and chestnut blight caused by *Cryphonectria parasitica* (Rigling and Prospero 2018). The proposed model is demo‐genetic, meaning it incorporates feedbacks mechanisms between the demography and genetic composition of the simulated pathogen population (Lamarins et al. 2022). The model is also spatially explicit, enabling the simulation of fine‐scale spatial heterogeneity in pathogen density, as well as realistic patterns of propagule dispersal across typical landscapes of cultivated perennial plants (Papaïx, Adamczyk‐Chauvat, et al. 2014; Papaïx, Touzeau, et al. 2014; Cunniffe et al. 2015; Lion and Gandon 2016; Thrall et al. 2016).

We used this integrated approach to explore how a mechanistic link between pathogen growth within plants and its spread among plants influences the relationship between the proportions of quantitatively resistant plants and the severity of epidemics caused by an introduced, evolving pathogen. We also aimed at providing predictive insights for leveraging resistances being screened for the pathosystems that inspired our model (e.g., Bauman et al. 2014; Stener 2018; Plumb et al. 2020). We specifically hypothesized that (i) a model linking within‐host pathogen multiplication and between‐host pathogen transmission would produce new epidemiological predictions contrasting with existing theory and (ii) the spatially explicit nature of the model would amplify the impact of this link on the outcomes. To test these hypotheses, we simulated multiple host landscapes with varying proportions of resistant hosts and differing resistance efficiencies. We compared the predictions of the full model with those of simpler versions where the pathogen load of infected plants did not influence disease transmission between plants, or the plant landscapes were non‐spatially explicit. We also simulated variations in pathogen biology and landscape management practices and analyzed the evolutionary trajectories of expanding pathogen populations through a quantitative genetics framework.

## 2 Methods

### 2.1 Host Landscape

The landscape simulated was a square matrix of $N_{h}$ host plants homogeneously distributed, such that the distance between contiguous plants was constant. The host simulated was a cultivated perennial plant (such as a fruit or forest tree, or a grape‐bearing vine) with a long lifetime spanning over multiple years. No natural recruitment occurred within the simulated stands. Each plant was represented by a set of K homogeneous compartments. The compartments represented different physical parts of the plants but were not spatialized. Each compartment could be infected by at most one pathogen genotype, but the compartments composing a plant could be infected by different pathogen genotypes, as frequently observed (Malpica et al. 2006). An infected compartment did not recover and host plants died only when all its compartments became infected (but see also ‘Alternative models’ paragraph below). Depending on the considered scenarios, dead hosts were either replaced by another identical healthy hosts or not replaced. Each host was characterized by Θ, a value reflecting its defense system against the pathogen. Θ could for instance integrate mechanical barriers, recognition of pathogen effectors and induced immune response, or any other components related to constitutive or induced defenses (Jones and Dangl 2006; Andersen et al. 2018). The environmental conditions were assumed to be constant and not affecting the plant defenses. The landscapes simulated included, in varying proportions, two classes of hosts either initially susceptible ($\Theta_{S}=0$) or resistant ($\Theta_{R}=6$) to the pathogen introduced (see Table 1 summarizing the scenarios investigated). We assumed that host resistance affected, in a similar way, both the probability of the host to be infected and the within‐host multiplication of the pathogen. In the landscapes, resistant hosts were deployed in continuous rows of $\sqrt{N_{h}}$ plants, evenly spaced, and the remaining space was occupied by susceptible hosts.

**TABLE 1:** Variable parameters in the simulations. Ninety‐six independent replicates were simulated for each scenario. The most common values used in the simulations are indicated in bold, where relevant.

Variable	Value
*Host landscape*	
Number of hosts $N_{h}$	**2500**, 10,000
Number of compartments K of each host	**20**, 40
Resistance efficiency (as %)	0, 30, 60, 89, > 99.99
Proportion of initially resistant hosts (as %)	0, 18, 36, 50, 67, 80, 100
Host initially infected	Susceptible, Resistant
Pathogen load *r* carried by the first infected host (as % of the number of compartments of each host)	5, 10, **20**, 30
Number of hosts initially infected	**1**, 2, 5
Replacement of entirely infected plants	Yes, **no**
*Fungal pathogen*	
Number of QTLs $N_{q}$	1, **10**
Heritability *h* ^*2*^ of infection strategy	0.4, **0.86**
Variance $\sigma_{Z}$ of infection strategy	1.5, **3**, 7
Number *x* of infectious spores resulting from sexual reproduction	**12**, 32
Probability *y* of survival after host death (in %)	**0**, 37, 75

### 2.2 Pathogen Virulence

**FIGURE 1:**
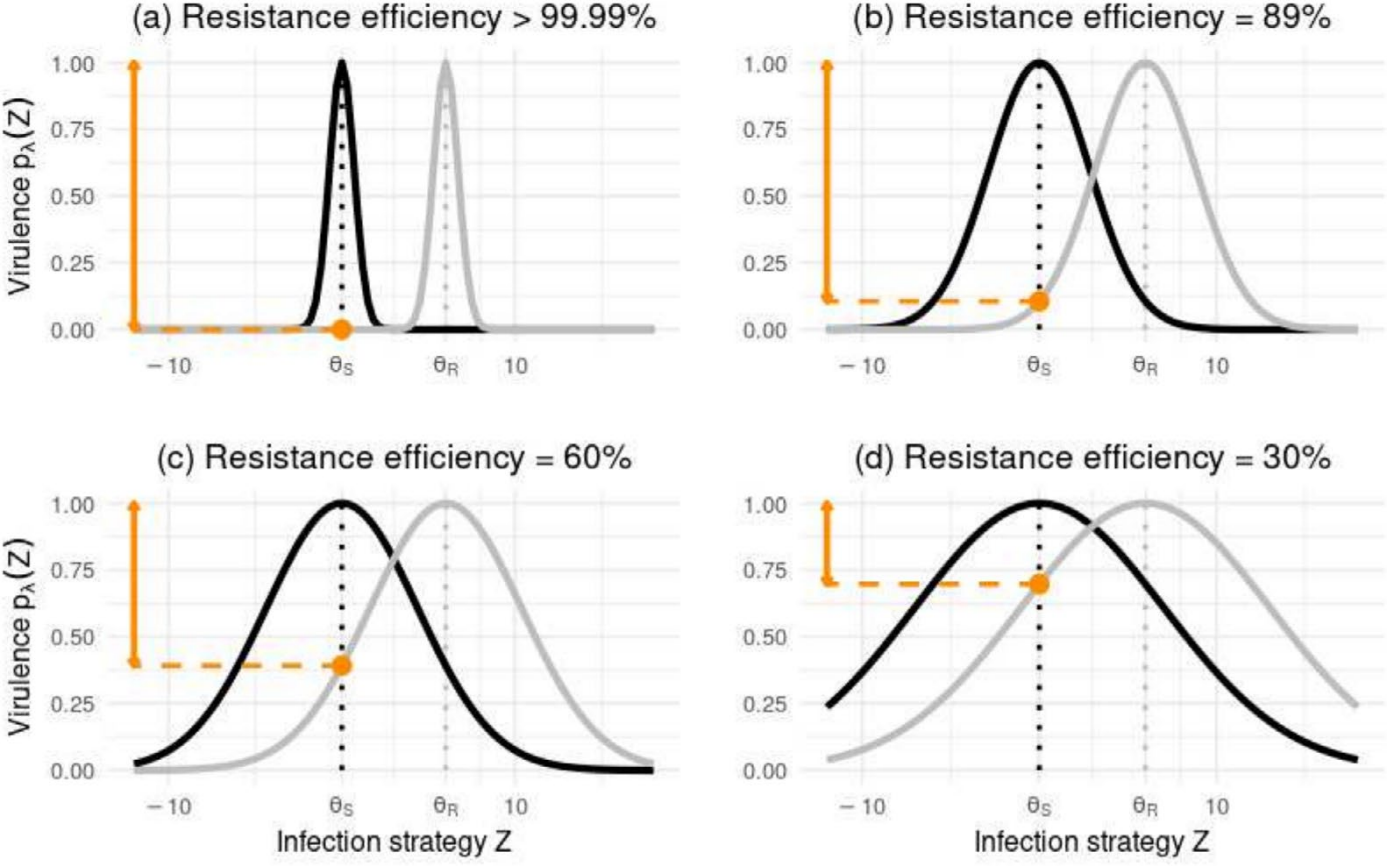
A quantitative relationship between the pathogen's infection strategy *Z* and its ability to infect and colonise initially susceptible (black curve) and resistant (gray curve) hosts. The dotted vertical lines represent the optimal trait values conferring the highest chance of infecting and colonizing susceptible ($x=\Theta_{S}$, black line) and resistant ($x=\Theta_{R}$, gray line) hosts, respectively. The introduced pathogen population consistently expressed a mean infection strategy value of 0. Four levels of resistance efficiency, defined by four distinct intensities of local stabilizing selection, were considered. These levels reduced the probability of infection by initial fungal genotypes by nearly 100% (a), 89% (b), 60% (c), and 30% (d) for resistant hosts. For each resistance efficiency, the fitness curves for susceptible and resistant hosts had the same width, but optimal infection strategy values differed. Therefore, any adaptation of the pathogen to one type of host (initially resistant or susceptible) implied a symmetrical maladaptation to the other type.

We call virulence the ability of the pathogen to initiate infections and to multiply within hosts. The virulence was determined by the interaction between infection strategy *Z* of the pathogen and Θ, the defense system of the host (Figure 1, see Equation 3 below). *Z* was assumed to be a quantitative trait that could for instance represent the set of effectors produced by a pathogen genotype in contact with a host (Lovelace et al. 2023). *Z* was heritable, and depended on the purely additive contribution of $N_{q}$ Quantitative Trait Loci (QTLs) recombining freely (recombination rate of 0.5). The trait value was determined from:

(1) $$Z=\sum_{i=1}^{N_{q}}\alpha_{i}+\epsilon$$

where $\alpha_{i}$ is the additive value of the allele carried at locus *i* and ϵ a micro‐environmental value drawn randomly for each infectious propagule from the Gaussian distribution *N*(0, $\sigma_{\epsilon }^{2}$). The heritability of the infection strategy was:

(2) $$h^{2}=\mathrm{Var}\left(\sum_{l=1}^{N_{q}}\alpha_{i}\right)/\mathrm{Var}\left(\sum_{l=1}^{N_{q}}\alpha_{i}+\epsilon \right)$$

Hence, for a constant phenotypic variance, the heritability of the trait decreased when $\sigma_{\epsilon }^{2}$ increased (see Table 1 for more information about the parametrization of the model).

The outcome of each infection attempt was stochastic and depended on both the pathogen genotype and Θ, the defenses of the host (Figure 1). A probability of infection $p_{\lambda }\left(Z\right)$, reflecting virulence, was calculated from the model of stabilizing selection (Turelli and Barton 1990):

(3) $$p_{\lambda }\left(Z\right)=\exp\left[\frac{-{\left(\Theta_{\lambda }-Z\right)}^{2}}{\omega^{2}}\right]$$

where $1/\omega^{2}$ is proportional to the strength of the local selection imposed by the host's defenses and λ designates the kind of host the pathogen encounters: susceptible (S) or resistant (R). The outcome of the infection attempt was then randomly determined from a Bernouilli law, weighted by $p_{\lambda }\left(Z\right)$. Hence, the closer the value of infection strategy expressed by the pathogen was to the local optimum defined by the host's defenses, the more probable the infection of a healthy compartment (Figure 1). The allelic effects $\alpha_{i}$ assigned to each of the $N_{q}$ alleles composing the initial fungal genotypes were drawn randomly and independently from the Gaussian distribution *N*($\Theta_{S}$, $\left(\sigma_{Z}^{2}-\sigma_{\epsilon }^{2}\right)/N_{q}$), where $\Theta_{S}$ was the optimal infection strategy value conferring the highest infection efficiency of susceptible plants by the initial pathogen genotypes (Figure 1). Hence, at the beginning of each simulation, the mean infection strategy value of the two genotypes introduced into the landscape tended to $\Theta_{S}$, resulting in genotypes that were well‐adapted to susceptible plants but maladapted to resistant plants. At the landscape scale, the difference between the defenses of resistant ($\Theta_{R}$) and susceptible ($\Theta_{S}$) hosts imposed divergent selection on the infection strategy of the pathogen, scaled by $1/\omega^{2}$. An important hypothesis underlying the model is that perfectly symmetrical fitness costs were assumed. Consequently, any pathogen evolution increasing the likelihood of infection of resistant hosts decreased symmetrically the likelihood of infection of susceptible hosts. The resistance efficiency (orange arrows in Figure 1) corresponded to the decrease for an introduced fungal pathogen, of the probability of infecting and colonizing a resistant host in comparison with the probability of infecting and colonizing a susceptible host ($\left|p_{S}\left(\Theta_{S}\right)-p_{R}\left(\Theta_{S}\right)\right|$ at cycle 1). The simulated quantitative resistance may reflect induced defense mechanisms, such as chemical or protein‐based responses (Eyles et al. 2010), or disease escape strategies, such as variation in leaf unfolding date (McKinney et al. 2011). Interestingly, variation in disease resistance has been associated with the expression of multiple genes in the pathosystems that inspired our model (e.g., Stocks et al. 2019; Westbrook et al. 2020). Overall, the host–pathogen interaction simulated here represents a polygenic and phenotype‐specific version of the matching‐allele model (Agrawal and Lively 2002) or of the gene‐for‐gene model with virulence costs (e.g., Leach et al. 2001). Note that the scaling of the resistance efficiency through $1/\omega^{2}$ (equation 3) made the intensity of stabilizing selection vary among the control landscapes (see the differences in the width of the black curves among the four panels of Figure 1).

### 2.3 Pathogen Life Cycle

The pathogen life cycle simulated was inspired by the biology of *Hymenoscyphus fraxineus*, the causal agent of Ash dieback in Europe, or *D. sapinea*, a pathogen of Scott pine. The fungus simulated was haploid, heterothallic, and thus reproduced sexually through fusions of genotypes bearing compatible gametes. The infectious propagules were the airborne spores, which are the results of meiosis events. Although capable of mycelial growth within infected plants, the pathogen did not produce clonal spores. Successive annual cycles of development were simulated, each cycle consisting of a sequence of eight steps:

1. *Production of gametes*. Each infectious fungal genotype established within a plant compartment produced two gametes, which were exact copies of the original genotype. We assumed this number to remain constant across genotypes and throughout the simulated cycles. However, at the plant scale, the number of gametes (and infectious propagules, see step 3 below) produced by an established fungal genotype depended on both the number of plant compartments it colonized and the lifespan of the infected plant.
2. *Gametes dispersal*. Each gamete was dispersed according to an ‘enhanced’ stepping stone dispersal model (Kimura and Weiss 1964). The direction of dispersal was randomly selected from eight possible directions (north, north–east, east, south–east, south, south–west, west, north–west). The distance of gamete dispersal was a positive number of intervals between contiguous plants, also drawn randomly from the Weibull distribution *W*(1, 0.3). The decimal part of each distance value drawn was systematically discarded. Thus, most gametes were dispersed on a plant scale. Dispersal was overall a fixed passive trait that did not evolve.
3. *Sexual reproduction*. The fungus being heterothallic, the mating type was determined by a single biallelic locus. This locus determined the possible pairs of parental genotypes: two genotypes could only fuse if their mating types differed. After dispersal, each gamete in contact with an infected plant could fertilize the sexual structure (ascogonium) of, at most, one established genotype of the opposite mating type. The compartment of the plant reached by the gamete was randomly drawn. When the gamete reached an infected compartment, fusion occurred (when the mating types differed) and *x* infectious spores were produced. The genotype carried by each infectious spore resulted from an independent meiosis. At the end of the reproduction step, all the gametes that did not fuse did not survive and were removed from the landscape.
4. *Aerial dispersal of infectious spores*. The propagules resulting from sexual reproduction were dispersed similarly to gametes (see step 2 above). The dispersal range of infectious spores was however much greater, as experimentally observed in most of Ascomycete fungi (e.g., Chandelier et al. 2014), and followed the Weibull distribution *W*(0, 3).
5. *Initiation of new infections*. Each year, healthy compartments of a plant were infected either by genotypes carried by infectious airborne spores that landed on the plant or by established pathogen genotypes spreading to new compartments through mycelial growth. In our simulations, disease transmission between plants corresponded to an infection of one plant caused by an airborne spore originating from another plant. Within‐plant pathogen multiplication resulted either from mycelial growth of established genotypes or landed spores originating from infected compartments of the same plant. The probability of compartment infection was determined by Equation (3) for each landed spore and established genotype. Hereafter, we use ‘infected compartments’ and ‘lesions’ interchangeably.
6. *Density regulation*. Each compartment of a plant could be infected by at most one pathogen genotype. For each plant, a density regulation step was realized when n, the number of new infections that could be induced by competing genotypes exceeded $K_{H}$, the number of healthy compartments. In such a case, $K_{H}-n$ of the new infectious genotypes were randomly removed and did not establish. Similarly, the genotype that did not induce new infections (Equation 3) did not survive and thus were removed from the landscape.
7. *Mutation*. The alleles at each locus mutated with a probability of 10^−5^ per year. Following the *k*‐allele mutation model (Peng et al. 2012), each mutated allele was replaced by another of the *k* = 256 possible alleles at the given locus. However, when a locus was associated with a mating type (see step 3 above), mutations remained silent as long as the number of mutations accumulated at this locus was lower than 100. When this threshold was exceeded, the mating type expressed was invalid and the genotype could no longer reproduce sexually.
8. *Death of the host and pathogen, virulence‐transmission trade‐off*. An established fungal genotype could undergo a maximum of 10 annual cycles on living hosts. Beyond these 10 cycles, the fungal genotype was removed from the landscape. When all compartments of a plant were infected, the plant died. Following this, all fungal genotypes that had colonized the plant also died unless the fungus was saprotroph (see below). Since the number of infectious propagules produced by each lesion was assumed constant in our simulations, the most virulent fungal genotypes could have a lower transmission than genotypes less adapted to their hosts, as largely hypothesized (Alizon et al. 2009). We also alleviated in some scenarios the virulence‐transmission trade‐off by simulating a fungus with saprotrophic capabilities. This ability allowed the fungus to complete up to three additional life cycles on the dead plant. During each of these additional cycles, a saprotrophic fungal genotype had a *y*% chance of surviving on the dead plant. Once the maximal number of cycles on a dead plant was exceeded, the fungal genotype died and was subsequently removed from the landscape.

### 2.4 Initialization of Simulations

We primarily examined situations in which a pathogen was introduced into the landscape through a single host carrying a low pathogen load. However, in some scenarios, we varied both the number of initially infected hosts and the initial magnitude of the pathogen load. Hence, at year 0 all hosts were healthy, except for one to five hosts colonized at rate *r* by two distinct fungal genotypes. Such a situation is typical of emerging diseases that inspired the model (e.g., McMullan et al. 2018). In each simulation, the first infected host was set in the upper‐left corner of the landscape. If multiple hosts were initially infected, the subsequent infections occurred sequentially in the host directly to the south, along the same row, at the western edge of the landscape. This contiguous pattern was adopted for enabling sexual reproduction between fungal genotypes established on adjacent hosts. This assumption of contiguity was critical for enabling the epidemic to develop when each infected host was colonized by a single fungal genotype (see step 3 above). The epidemics could start either from a susceptible or a resistant host. Depending on the scenario, the row at the western edge of the landscape was assumed to be composed of susceptible or resistant hosts.

$\sigma_{Z}^{2}$, the maximal variance of the pathogen's infection strategy was set to $\left|\Theta_{R}-\Theta_{S}\right|/2$. The initial proportion of the mating types assigned to the initial pathogen genotypes was set even, so that sexual reproduction and epidemic development could occur all else being equal.

We simulated seven contrasting landscapes. In the control landscape, all hosts were susceptible to the pathogen, defining a single optimal value of the infection strategy at the landscape scale corresponding to $\Theta_{S}$. In the other simulated landscapes, different proportions of resistant hosts were introduced, ranging from 18% to 100%.

We compared deployment scenarios involving different quantitative resistance efficiencies: a nearly complete resistance (protection against introduced genotypes greater than 99.99%), a strong resistance (89% efficient), a moderate resistance (60% efficient) and a weak resistance (30% efficient) (Figure 1). These four distinct resistance efficiencies were obtained by simulating four different strengths of local stabilizing selection, at the landscape scale (Equation 3, see the distinct values of ω in Table 1). For the sake of simplicity, in what follows, the quantitative resistance with an efficiency greater than 99.99% is called ‘nearly complete’, while other resistances are called ‘partial’. Several other variables of the model were varied, related to the evolutionary potential of the pathogen (number of quantitative trait loci involved in the expression of the infection strategy, variance of the allelic effects defining the space of possible infection strategy values, heritability of the infection strategy), and its ability of transmission (number of infectious propagules produced by lesions). In the main scenarios investigated, dead hosts were not replaced, and the pathogen died as its host died. We also considered scenarios where entirely infected hosts were replaced by new healthy ones, and scenarios where the pathogen was saprotrophic (see step 8 above). Ninety‐six independent replicates were simulated for each scenario. Each replicate was run over 100 annual cycles. In most scenarios, disease emergence was followed by an epidemic peak and pathogen disappearance within a span of less than 50 years. The simulated pathogen life cycle was assumed to be annual; however, our predictions could also apply to pathosystems in which the pathogen undergoes multiple reproductive cycles within a single year. Table 1 provides a summary of the explorations of the model.

### 2.5 Monitoring the Severity of Epidemics and the Evolution of Pathogens

The severity of epidemics was assessed from three key indicators: the proportion of surviving hosts, the total number of healthy compartments and the cumulative number of infections across the landscape. We also tracked the total number of active lesions during the simulations, which informed us about changes in the intensity of the inoculum pressure at the landscape scale. Additionally, we recorded the mean and variance of the infection strategy of the established pathogen genotypes. The degree of genetic differentiation for the infection strategy between the genotypes established on susceptible and resistant hosts was monitored through $Q_{\mathrm{ST}}$ measures (Spitze 1993). $Q_{\mathrm{ST}}$ is a widely used measure of genetic differentiation linked to a quantitative trait. It is computed from the additive genetic values observed in the populations. In contrast to $F_{\mathrm{ST}}$ values, which are calculated individually for each QTL, $Q_{\mathrm{ST}}$ takes into account the covariance among all QTLs contributing to the trait's variability (Le Corre and Kremer 2012). In our simulation, the $Q_{\mathrm{ST}}$ values were directly calculated from $V_{B}$, the variance of the mean trait value between the two populations, and $V_{W}$ the mean of the variance of the trait value within each population:

(4) $$Q_{\mathrm{ST}}=\frac{V_{B}}{\left(V_{B}+V_{W}\right)}$$

### 2.6 Alternative Models

We evaluated the influence of model structure on epidemiological outcomes by comparing the predictions of two simplified models against those generated by the main model described above. The first alternative model relaxed the mechanistic link between within‐ and between‐plants disease transmission. Therefore in this version, the contribution of infected plants to disease transmission was independent of their pathogen load. To achieve this, the lifetime of infected plants was fixed at 3 years, regardless of the number of infected compartments. In addition, the total number of gametes (step 1 in the pathogen life cycle) and infectious propagules (step 3) produced by each infected plant was also standardized to 2 and 16, respectively. With these values, the epidemic severity predicted by the alternative model in landscapes composed entirely of susceptible plants was quantitatively comparable to that predicted by the complete model. Note that the production of infectious propagules in this simplified model still required sexual reproduction. A random density regulation was applied when the total number of gametes or infectious propagules produced by the infectious fungal genotypes of a plant exceeded the fixed threshold. Conversely, when the initial total of infectious propagules was too low, the number of propagules per sexual reproduction event was increased to meet this threshold (the number of gametes per infected plant was always at least two).

The second simplified version of the model was not spatially explicit. In this version, susceptible and resistant plants were randomly distributed, with a new landscape generated randomly for each simulation replicate. In addition, each gamete (step 2 in the pathogen life cycle) and infectious propagule (step 4) could reach any plant in the landscape with equal probability. In all the additional simulations using alternative versions of the model, the first infected host was resistant.

## 3 Results

### 3.1 Epidemic Development

**FIGURE 2:**
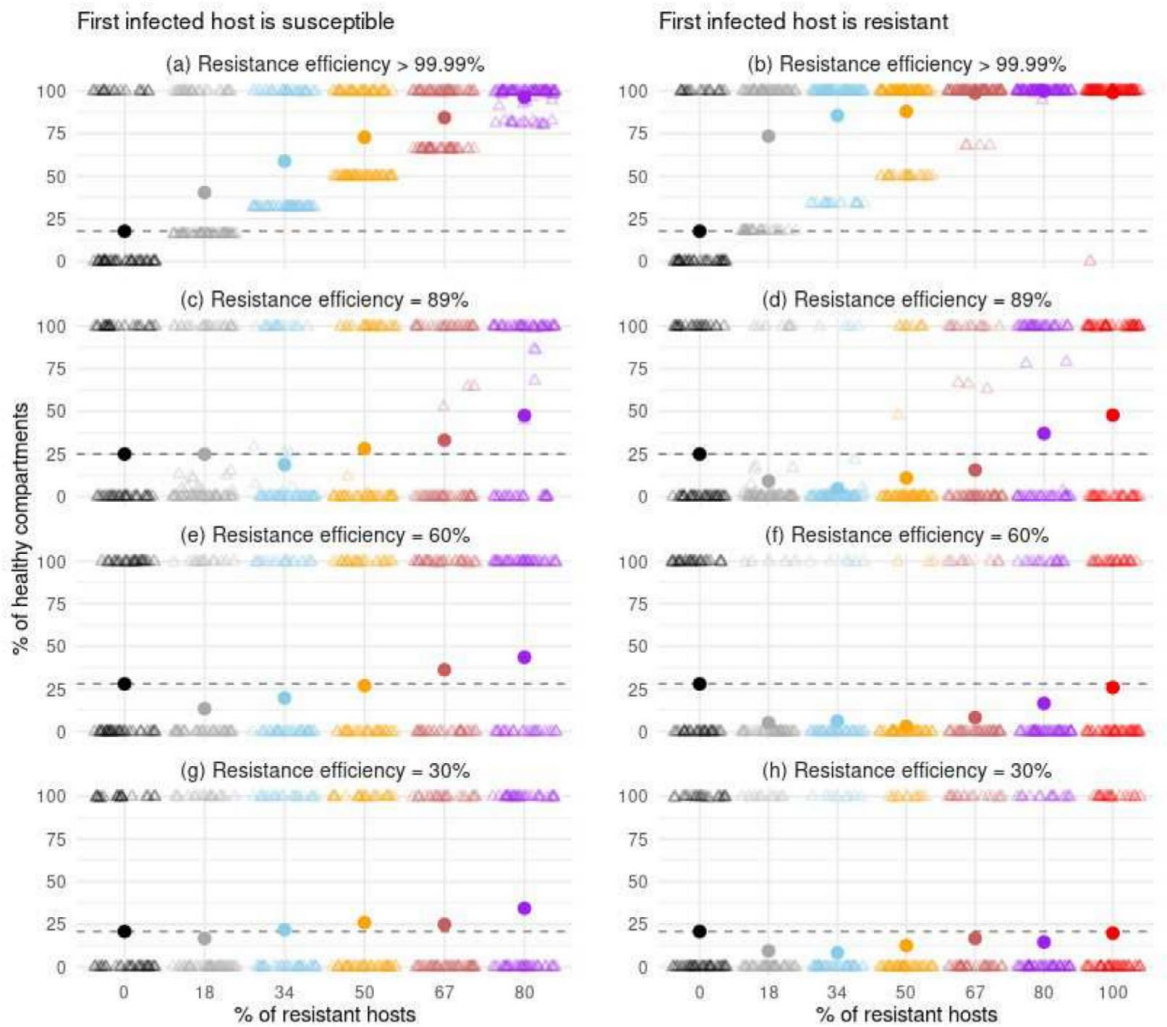
Percentage of healthy compartments, at equilibrium, as a function of the proportion of resistant hosts in the landscape and the efficiency of resistance. In these scenarios, dead host plants were left unreplaced. The first infected host was either susceptible (left column) or resistant (right column). Each point represents the mean of 96 replicates of a scenario. Each light triangle indicates the percentage of infected hosts within a single replicate.

**FIGURE 3:**
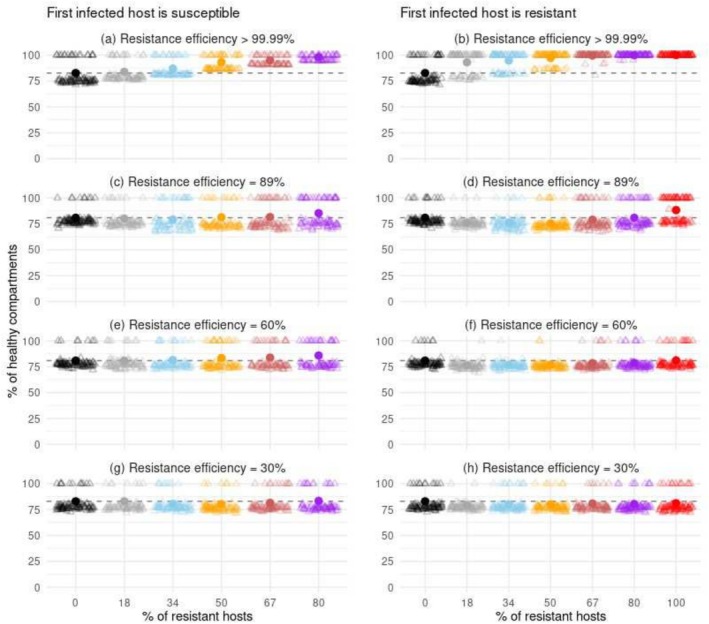
Percentage of healthy compartments, at equilibrium, as a function of the proportion of resistant hosts in the landscape and the efficiency of resistance. Here dead plants were systematically replaced by healthy ones. The first infected plant was either susceptible (left column) or resistant (right column). Each point represents the mean of 96 replicates of a scenario. Each light triangle indicates the percentage of infected hosts within a single replicate.

**FIGURE 4:**
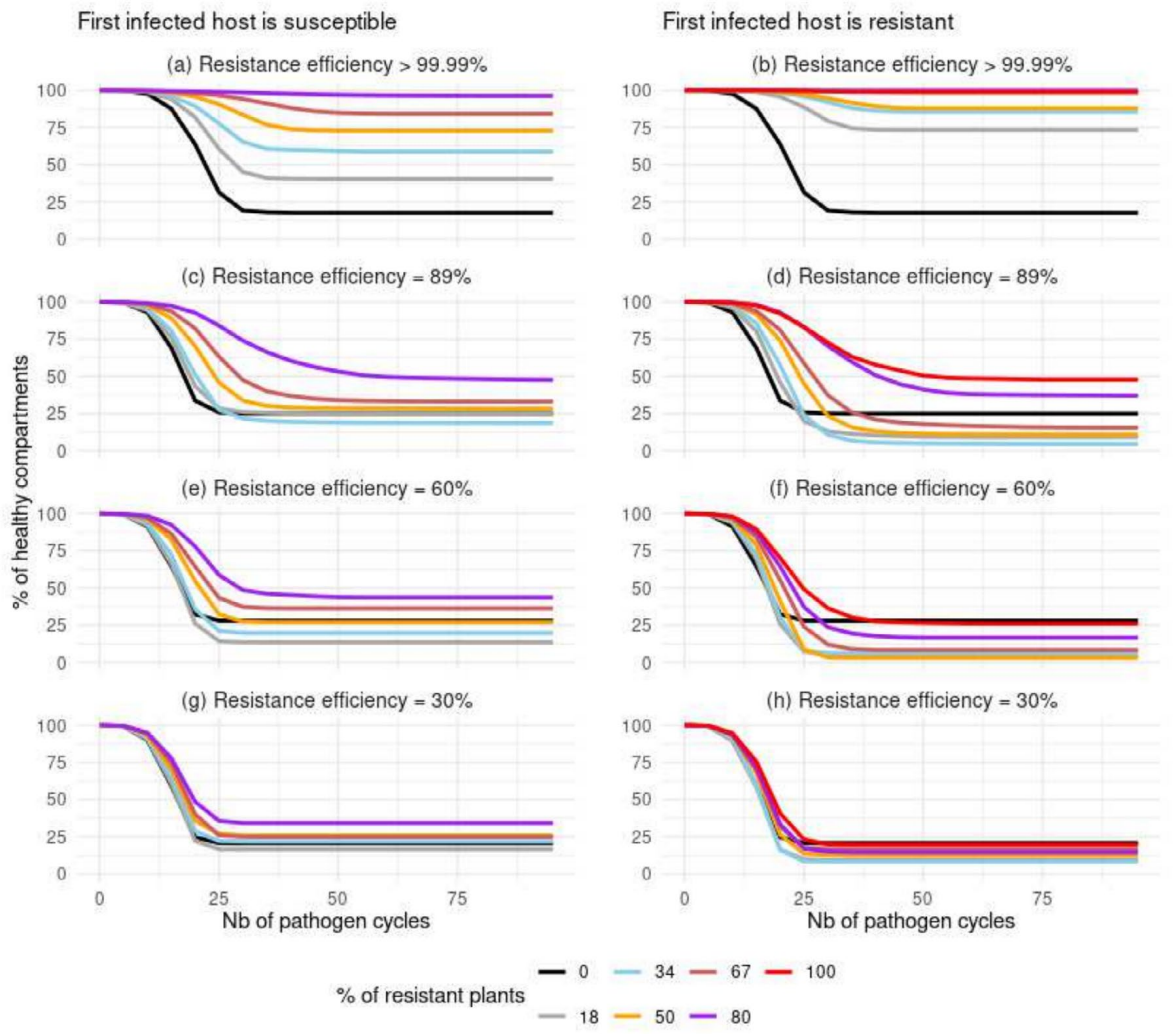
Proportion of healthy compartments over the years since pathogen introduction. The first infected plant was either susceptible (left column) or resistant (right column). In these scenarios, dead plants were left unreplaced. Each line denotes the mean of 96 replicates of a scenario.

The introduction of infected plants resulted in either the spread of the disease or the extinction of the pathogen population. This bimodal distribution of epidemiological outcomes was observed in most scenarios at equilibrium across the 96 simulated replicates (see the light triangles in Figures 2 and 3). Specifically, in scenarios without dead plant replacement and in landscapes without nearly completely resistant plants, two primary outcomes were observed: the pathogen population either rapidly went extinct, or it infected and subsequently killed all hosts. Following the successful introduction of the pathogen, there was a logistic decrease in both the total proportion of healthy compartments and the percentage of surviving hosts (Figure 4 and Figure S1). Concurrently, the number of infected hosts increased symmetrically (Figure S2). Given the consistent impact of resistance deployment on healthy compartments, infection rates, and mortality, our subsequent analysis focused primarily on the percentage of healthy compartments. This metric served as an indicator of epidemic impact and severity. Across all scenarios, the onset of epidemics showed a continuous pattern of propagation, originating from the upper‐left corner of the landscape (Figure S3).

### 3.2 The Use of Nearly Complete Resistance Efficiently Prevented Epidemic Development

The deployment of a nearly complete resistance consistently reduced epidemic damages, both over the short and long terms. The protection conferred was most effective when the pathogen was introduced into the landscape through a resistant host (Figures 2 and 4 panel b). In such cases, the relationship between the proportion of resistant hosts and the mean proportion of healthy compartments at equilibrium was logistic, with a plateau of 100% healthy compartments being rapidly reached. For instance, 72% of the compartments remained healthy when only 18% of the hosts were resistant, compared to just 17% in a completely susceptible landscape. In contrast, when the first infected host was susceptible, the epidemic severity exhibited a more linear response to the number of resistant hosts (Figures 2 and 4, panel a). Obtaining a survival rate of approximately 72% for the hosts' compartments required 50% of the hosts to be resistant.

The use of a nearly complete resistance consistently prevented epidemic development in simulations based on the simplified versions of the model, in which within‐plant pathogen multiplication and between‐plant transmission were uncoupled, or the landscape description was non‐spatially explicit (Figure S4, panels a and b).

### 3.3 The Use of Partial Resistance Could Increase Epidemic Severity

The deployment of partial resistances mostly reduced epidemic damages over the short‐to‐medium term. This protective effect was limited when the resistance used was weak, but strengthened with the efficiency of the resistance and the proportion of resistant hosts (Figure 4, panels c–h). However, over the long term, the severity of the epidemic was similar or sometimes worse in landscapes with partially resistant hosts than in completely susceptible landscapes (Figures 2 and 4, panels c–h). This trend was most evident when the first infected host was resistant, the proportion of resistant hosts was lower than 80%, and the resistance efficiency ranged from moderate to strong. Interestingly, epidemic severity slightly worsened when the first infected host was susceptible and 18% or 34% of the plants were moderately resistant to the pathogen (Figure 2, panel e).

Increases in disease severity following resistance deployment were still observed over the long term using a non‐spatially explicit model, but only when the proportion of resistant hosts was lower or equal than 34% (Figure S4, panels c and e). Constrastingly, when the contribution of infected plants to disease transmission did not depend on the pathogen load they carried, partial resistance deployment consistently provided long‐term protective effects, with higher proportions of resistant plants offering the greatest protection against disease progression (Figure S4, right column).

Overall, the counter‐intuitive prediction of increased epidemic severity following partial resistance deployment provided by our main model held as long as epidemic development was not systematic in entirely susceptible landscapes (Figures S5–S8). Hence, the deployment of a moderate resistance also increased host mortality when the number of hosts was increased, or when the heritability, the possible variability of the pathogen's infection strategy, or the number of QTLs determining the trait value were decreased. Doubling the quantity of infectious propagules produced after each sexual reproduction event also led to the same qualitative prediction, although the differences observed among the scenarios were reduced (Figure S6). Interestingly, replacing dead plants with healthy ones led to fluctuations in epidemic damage over the years (light lines in Figure S9). In these scenarios, the reintroduction of healthy plants sustained the expanding pathogen population, and epidemic severity was also slightly higher in several landscapes in which partial resistances were deployed than in entirely susceptible landscapes (Figure 3, panels d, f, g, h). In scenarios without dead plant replacement, epidemic development was almost systematic when the pathogen simulated was highly saprotrophic (i.e., had a good probability to persist on dead plant tissues). In such a case, partial resistance deployment had no influence on host mortality over the long term (Figure S7a). Yet, saprotrophism can be less efficient, or dead plant replacement can recurrently remove part of the saprotrophic fungal genotypes. In these scenarios, we observed slight epidemic intensifications associated with partial resistance deployment (Figure S7, panels b and c). Besides, the level and spatial distribution of the initial pathogen load affected the probability of sexual reproduction, and thus, the production of infectious propagules in the early stages of the epidemics. Nevertheless, across most simulated patterns of pathogen introduction, we observed landscapes with partially resistant hosts that underwent slightly more epidemic damages than entirely susceptible landscapes (Figure S8).

### 3.4 The Deployment of Partial Resistance May Increase the Overall Inoculum Pressure

**FIGURE 5:**
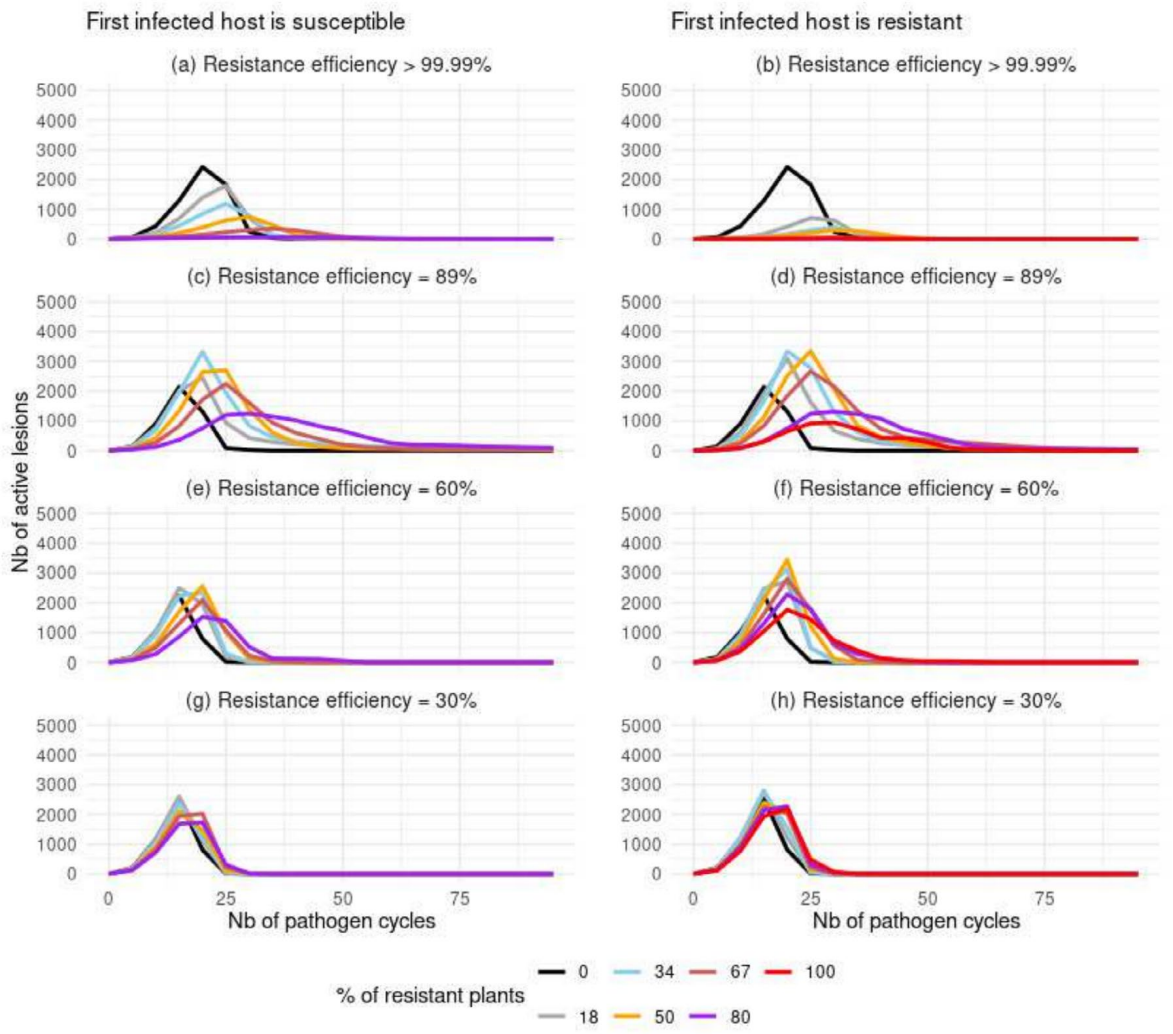
Mean number of active lesions as a function of time elapsed since pathogen introduction. The first infected plant was either susceptible (left column) or resistant (right column). Each line is the mean of 96 replicates of a scenario, involving a given proportion of resistant plants in the landscape.

In all scenarios of nearly complete resistance deployment, the proportion of resistant plants infected was consistently very low (< 0.01%, Figure S10, panels a and b). This low infection rate reduced the maximal peak of active lesions (Figure 5, panels a and b). The likelihood of resistant plants becoming infected increased as the initial efficiency of the deployed resistance decreased (Equation 3, see also Figure S10, panels c–h). Nonetheless, across all scenarios of partial resistance deployment, the number of infected compartments increased more rapidly in susceptible plants than in resistant ones (Figures S10 and S11). Paradoxically, the increase in epidemic severity following the deployment of a partial resistance was concomitant with a transiently lower mortality rate of infected hosts compared to entirely susceptible landscapes. For instance, at year 25, the ratio of killed plants to infected plants approached 1 in entirely susceptible populations, whereas it approximated 0.77 when half of the plants were strongly resistant (computed from black and orange lines in Figures S10 and S11, panel d). In this context, the deployment of partial resistance delayed the maximal peak of the number of active lesions (Figure 5). Interestingly, in some scenarios of partial resistance deployment, this delay was accompanied by an increase in the production of infectious spores (Figure 5, e.g., orange lines), which ultimately led to an increase in epidemic severity. Conversely, a reduction in epidemic severity due to resistance deployment was associated with a decrease in the maximal peak of active lesions (Figure 5, e.g., red lines in panels b, d, f).

### 3.5 Evolution of Pathogen's Infection Strategy

**FIGURE 6:**
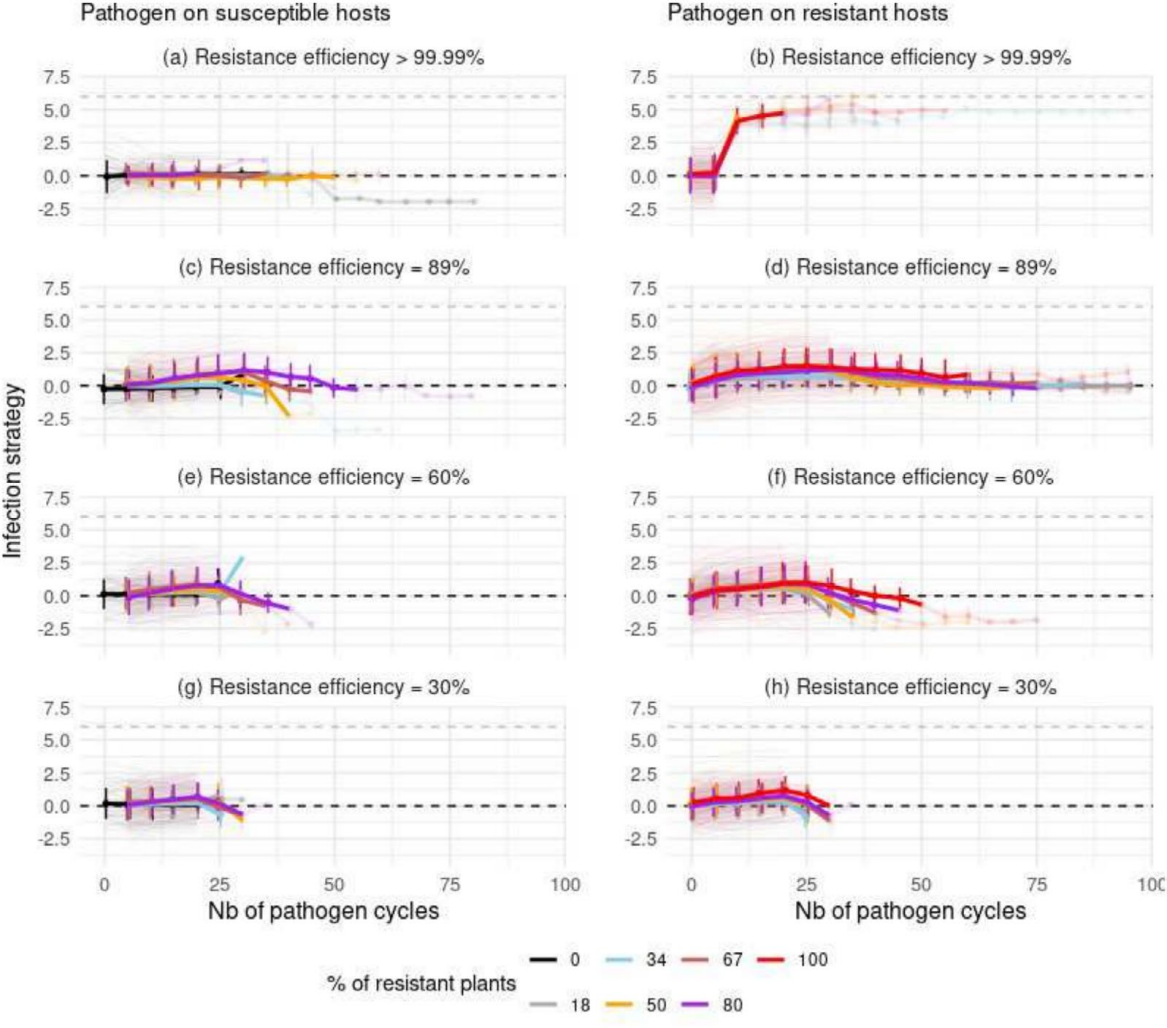
Mean infection strategy value of pathogen populations established on susceptible hosts (left column) and resistant hosts (right column), tracked over the years following pathogen introduction. In this scenario, the first infected host was resistant. The thick lines represent the mean infection strategy values calculated across 96 replicates of a scenario. Each bar indicates the standard deviation of the corresponding mean value. Each light line represents the infection strategy within a single replicate. The opacity of the thick lines and bars was reduced when the number of non‐null replicates fell below 10. The black and gray dashed lines correspond to the optimal infection strategy values that confer the highest chance of infecting susceptible ($\Theta_{S}=0$) and resistant hosts ($\Theta_{R}=6$), respectively.

Without resistance in the landscape, the mean value of pathogen infection strategy remained, at the epidemic peak (year 20 on average, see Figure 5), close to $\Theta_{S}$, the optimal value that conferred to the pathogen the highest chance of infection of susceptible hosts (Figure 6 and Figure S12, black lines). In contrast, the deployment of resistances induced a rapid change in the mean infection strategy expressed by the pathogen, according to two main patterns, determined by the efficiency of the resistance used (Figure 6 and Figure S12, coloured lines).

In landscapes in which nearly complete resistances were deployed, the few pathogen genotypes that successfully settled on resistant hosts rapidly expressed an infection strategy approaching $\Theta_{R}$, the optimal value allowing the pathogen to overwhelm the defenses of resistant hosts (Figure 6 panel b). Contrastingly, the genotypes established on susceptible hosts expressed an infection strategy value of $\Theta_{S}$ (Figure 6, panel a). This differentiation was associated with a drop in the additive variance within pathogen populations (Figure S13), resulting in a strong genetic differentiation between the genotypes established on susceptible hosts and those established on resistant hosts (Figure S14, panels a and b, see also Figure S15). Although the infection strategy expressed by the pathogen on resistant hosts was never optimal, the distribution of infection strategy values at equilibrium was bimodal in this case. This indicates that the pathogen population consisted of a coalition of specialist genotypes, each specifically infecting either resistant or susceptible hosts.

The deployment of partial resistances caused the infection strategy to slightly evolve towards a value closer to optimal value $\Theta_{R}$ (Figure 6 and Figure S12, panels c–h). In this case, the genetic differentiation generated was low (Figure S14, panels c–h). The distribution of the pathogen's infection strategy values remained unimodal and the pathogen population consisted of genotypes that infected both resistant and susceptible hosts. Compared to the genotypes initially introduced, these evolved genotypes were less adapted to susceptible hosts but better adapted to resistant hosts. This evolution pattern consisting in ‘intermediate’ infection strategy principally prevailed when the first plant infected was resistant (compare Figure 6 and Figure S12, panels c–h).

The change in the infection strategy expressed by the pathogen on partially resistant hosts was the fastest and the largest when the first infected plant was resistant and the resistance efficiency was high (Figure 6). This rapid change is a consequence of directional selection pressure imposed on the pathogen's infection strategy at the host level during the onset of the epidemic. Following the initial increase observed on partially resistant hosts in most scenarios, there was a subsequent decrease in infection strategy values towards $\Theta_{S}$. This suggests that the rare infections of resistant hosts in the later stages of epidemic development were likely caused by maladapted genotypes, originating either from susceptible hosts or established for a long time. Note that in several scenarios, the very limited size of the fungal population after year 30 (see Figure 5) led to stochastic variations in the infection strategy (see also the very low number of light curves after year 30 in Figure 6).

## 4 Discussion

### 4.1 Findings

We proposed a new spatially explicit demo‐genetic model of quantitative resistance deployment in managed stands of perennial plants. The model mechanistically couples pathogen multiplication within plants and its spread among them. It is grounded in quantitative genetics and based on the assumption that pathogen adaptation to resistant hosts results in reciprocal maladaptation to susceptible hosts. Consistent with existing theory, our simulations confirm that deploying nearly complete resistance can effectively and sustainably protect host plants against introduced fungal pathogens. However, our model also yields counterintuitive predictions, where the use of partial resistance increases epidemic severity compared to landscapes composed entirely of susceptible plants. This phenomenon occurred in our simulations primarily because the maladaptation of infectious genotypes to their hosts prolonged the lifespan of infected hosts, allowing the pathogen to sporulate longer. In certain situations, the evolution of infection strategy resulted in pathogen maladaptation to both susceptible and resistant hosts, leading to a global increase in inoculum pressure and, consequently, disease severity (see details in the next paragraph). The likelihood that resistance deployment increases epidemic severity was highest under the following conditions: (i) the use of resistance with strong (89%) or moderate (60%) efficiency in intermediate proportions, (ii) pathogen spread originating from a resistant host and (iii) development of epidemics unlikely in entirely susceptible landscapes due to the biological characteristics of the pathosystem, its management, or the pattern of pathogen introduction. This outcome was particularly evident in our spatially explicit simulations where resistant plants were deployed in rows, but it was also observed in the non‐spatially explicit version of the model. However, no increase in epidemic severity was observed in simulations where within‐plant pathogen growth and between‐plant spread were decoupled, highlighting the influence of the latter component on model predictions.

Many modeling studies focusing on agricultural landscapes and accounting for pathogen evolution have identified optimal strategies for resistance deployment, from both evolutionary and epidemiological perspectives (Zhan et al. 2015; Rimbaud et al. 2021). Nevertheless, to the best of our knowledge, no existing model assuming adaptation costs for the pathogen has predicted that the use of partial resistance could increase epidemic severity in plant populations. While the model by van den Berg et al. (2014) focused on quantitative resistance in plants, and other models inspired by insect diseases (De Roode et al. 2011) or imperfect vaccines in humans (e.g., Gandon and Michalakis 2000; Gandon et al. 2001; Mackinnon et al. 2008), have predicted exacerbation of virulence following partial resistance deployment, these predictions were made without assuming pathogen adaptation costs. In addition, the resistances in these models sometimes targeted different pathogen life‐history traits. Thus, the mechanisms driving higher virulence in these studies, through competing interactions among pathogen strains, are not directly applicable to our context. For instance, van den Berg et al. (2014) modeled a resistance that reduced pathogen sporulation per lesion unit, indirectly selecting for higher virulence through the evolution of larger lesion sizes. Similarly, De Roode et al. (2011) predicted that a resistance decreasing the pathogen load during infection initiations could select for higher whithin‐host multiplication leading to higher virulence. Gandon and Michalakis (2000) and Gandon et al. (2001) (see also Mackinnon et al. 2008) showed that partial resistances targeting within‐host pathogen multiplication could drive the evolution of higher virulence levels by reducing the negative impact of high virulence on disease transmission. In contrast, these two later models also suggested that partial resistance reducing infection likelihood (as in our study) protect populations by limiting competition among pathogen strains and ultimately constraining virulence evolution. This latter prediction contrasts sharply with our findings. Hence, our work extends existing theory by demonstrating that resistances partially preventing infections, while imposing pathogen adaptation costs, can paradoxically increase epidemic severity in perennial organisms.

Among the scenarios of resistance deployment, landscapes with the highest proportions of resistant hosts typically yielded the greatest host survival rates. This result aligns with predictions from many resistance deployment models applied to well‐mixed landscapes that exclude pathogen evolution (e.g., Holt and Chancellor 1999; Skelsey et al. 2010; Papaïx, Adamczyk‐Chauvat, et al. 2014; Papaïx, Touzeau, et al. 2014). It also supports predictions from models of quantitative resistance deployment that account for pathogen evolution, such as those by Gandon et al. (2001), Iacono et al. (2012) and Fabre et al. (2022). Our result further substantiates the idea that a high proportion of resistant hosts imposes strong demographic constraints on the pathogen population, thereby reducing both the likelihood of disease emergence and the potential for pathogen adaptation. In contrast, other models of quantitative (e.g., Chabas et al. 2018; Papaïx et al. 2018) and qualitative (e.g., Ohtsuki and Sasaki 2006; Fabre et al. 2012; Rimbaud et al. 2018; Saubin et al. 2021) resistances suggest that intermediate proportions of resistant hosts in the landscape may optimize long‐term epidemiological control by alleviating the intensity of the selection pressure on the pathogen's infection strategy. These latter predictions echoes findings from many modeling studies on fungicides, which suggest that reducing pathogen exposure to treatment lowers the likelihood of pathogen adaptation, thereby potentially maximizing long‐term epidemiological control (e.g., van den Bosch et al. 2014; Carolan et al. 2017; Taylor and Cunniffe 2023). Overall, these predictions stand in sharp contrast to our findings. Major differences between these models and ours include the absence of a mechanistic interdependence between pathogen multiplication within hosts and its transmission among hosts (Tellioglu et al. 2022; Jiranek et al. 2023), no spatially explicit description at the fine scale of the host level (Thrall et al. 2016), and the absence of explicit interactions among pathogen strains at the host level (Lamarins et al. 2022).

The increased epidemic severity associated with the deployment of partial resistance coincided with limited pathogen evolution. In some scenarios, this evolution primarily resulted in substantial maladaptation of the pathogen to both susceptible and resistant hosts. Conversely, the deployment of nearly complete resistance was associated with a higher level of pathogen adaptation to the host. Hence, akin to extant theory on the evolution of polymorphisms, we found that the strength of selection determined the distribution of infection strategy values at equilibrium. The strongest selection pressures led to bimodal distributions of trait values, suggestive of a certain degree of specialization of pathogen genotypes to each type of host. In contrast, weaker selection pressures resulted in unimodal distributions of non‐optimal infection strategy values, more typical of the prevalence of generalist genotypes (Ravigné et al. 2004). These evolutionary equilibria are consistent with the predictions of models of population expansion over heterogeneous landscapes with varying levels of migration and strengths of selection (Day 2000; Ronce and Kirkpatrick 2001; Débarre et al. 2013). They also align with predictions of evolutionary equilibria produced by the models of quantitative resistance deployment proposed by Gudelj et al. (2004) and Fabre et al. (2022).

### 4.2 Partial Resistances and Pathogen Evolution Can Increase Pathogen's Transmission

Assuming a quantitative host–pathogen interaction, a certain degree of maladaptation of the pathogen to its host decreases the frequency of new infections without completely preventing them. In our simulations, hosts infected by maladapted genotypes survived, on average, longer than hosts colonized by perfectly adapted genotypes, as observed in entirely susceptible landscapes or in some scenarios of strong resistance deployment. By decreasing the pathogen's fitness, partial resistance extended the period of infectious spores production by the genotypes they were infected by. Under certain conditions, this could amplify the inoculum pressure at the landscape scale. Interestingly, this mechanism echoes the findings of theoretical studies that indicate the optimal level of virulence (a consequence of the pathogen's adaptation to its host) is often intermediate and depends on the interaction between disease transmission and disease‐induced mortality (the ‘virulence transmission trade‐off’, May and Anderson 1983; Day 2001; Gilchrist and Coombs 2006; Alizon et al. 2009). However, the amplification the overall inoculum pressure through resistance deployment can only occur when (i) the overall degree of maladaptation of established pathogen genotypes is intermediate and (ii) a sufficient number of pathogen genotypes have colonized hosts to which they are maladapted. In our simulations these conditions were most easily met when the first infected host was resistant, and the deployed resistance was moderate or strong. In such circumstances, the extended lifetime of the first infected hosts, coupled with the rapid prevalence of maladapted genotypes in the pathogen population, sustained epidemic development from its onset. Interestingly, these conditions were sometimes met when the first infected host was susceptible, particularly when the proportion of resistant hosts in the landscape was low. In this scenario, the high availability of susceptible hosts in the landscape allowed a steady development of the pathogen population following its introduction. Once the inoculum pressure exceeded a certain threshold, some resistant hosts became infected and were slowly colonized by maladapted pathogen genotypes, which in turn further amplified the inoculum pressure and increased host mortality. Using weak resistances alleviated this effect, due to the limited level of maladaptation inherent to the weak selection pressures simulated in this case. When the resistance deployed was almost complete, the degree of specialization of the fungal genotypes to their host was higher. In this case, the resistant hosts deployed primarily acted as spore sinks (Holt and Barfield 2011), and the pathogen load carried by strongly resistant hosts remained too low to affect the overall inoculum pressure.

## 5 Conclusion and Perspectives

Further work is necessary to clarify the conditions under which partial resistance deployment can amplify disease severity. Specifically, although we considered both fixed and randomly generated landscapes, additional investigations are needed to assess the influence of the spatial distribution of resistant plants on our predictions (Xu and Ridout 2000; Skelsey et al. 2010; Sapoukhina et al. 2013; Papaïx et al. 2018). The applicability of this prediction to pathogens with different life cycles, such as those involving clonal reproduction during plant‐to‐plant transmission, should also be examined (Brown 1995; Bazin et al. 2014). In addition, accounting for plant recovery and the potential for reinfections of recovered compartments is necessary. Finally, exploring how a mechanistic interdependence between within‐ and between‐plant disease transmission shapes host–pathogen co‐evolution dynamics would provide valuable insights applicable to plant populations where natural recruitment occurs (e.g., Boots and Haraguchi 1999; Hulse et al. 2023).

Nevertheless, our study calls for caution in the deployment of partially effective resistances. Our prediction of increased epidemic severity associated with resistance deployments adds to the findings by Gandon and Michalakis (2000), Gandon et al. (2001), De Roode et al. (2011) and van den Berg et al. (2014), who highlighted different mechanisms leading to a similar outcome. These results underscore the importance of optimizing yield or host survival by developing a thorough understanding of the molecular mechanisms underlying plant–pathogen interactions (Agrawal and Lively 2002). This includes quantitative assessments of resistance efficiency against a range of pathogen strains that could be introduced as well as evaluations of the pathogen's dispersal capabilities (see also the recommendations by Saubin et al. 2023). According to our model, the risk of disease amplification through the deployment of partial resistance is considerable when disease‐induced mortality is moderate in entirely susceptible host populations. This risk can arise as a direct consequence of landscape fragmentation or pathogen's biology. Furthermore, this risk is largely increased when the pathogen is introduced into the host population via a resistant host. This finding underscores the critical need for systematic health monitoring of partially resistant plants introduced into exploited stands.

## Conflicts of Interest

The authors declare no conflicts of interest.

## Supporting information


**Figure S1.** Percentage of living hosts over the years since the pathogen introduction.
**Figure S2.** Percentage of infected hosts over years, since the pathogen introduction.
**Figure S3.** Epidemic development following pathogen introduction.
**Figure S4.** Percentage of healthy compartments over time since pathogen introduction.
**Figure S5.** Percentage of healthy compartements, at equilibrium, as a function of the percentage of resistant hosts in the landscape.
**Figure S6.** Percentage of healthy compartments, at equilibrium, as a function of the percentage of resistant hosts in the landscape, in additionnal scenarios.
**Figure S7.** Percentage of healthy compartments, at equilibrium, as a function of the percentage of resistant hosts in the landscape, in additional scenarios involving a saprotrophic pathogen.
**Figure S8.** Percentage of healthy compartments, at equilibrium, as a function of the percentage of resistant hosts in the landscape, in additional scenarios.
**Figure S9.** Proportion of healthy compartments over the years since pathogen introduction.
**Figure S10.** Number of resistant healthy compartments over time following pathogen introduction.
**Figure S11.** Number of susceptible healthy compartments over time following pathogen introduction.
**Figure S12.** Mean infection strategy value of pathogen populations established on susceptible (left column) and resistant hosts (right column), over years following pathogen introduction.
**Figure S13.** Mean variance (*Vw*) of infection strategy values within pathogen populations established on susceptible and resistant hosts.
**Figure S14.** Genetic differentiation (*QST*) for infection strategy at year 20, between the population of pathogen established on susceptible hosts and the one established on resistant hosts.
**Figure S15.** Variance (*Vb*) of mean infection strategy values of the pathogen populations established on susceptible and resistant hosts.

## Data Availability

The code and simulated data supporting the findings of this study are openly available at https://doi.org/10.57745/LSMX6G (Soularue 2025).
