# Supplementary material for: Quantitative Resistance Deployment Can Strengthen Epidemics in Perennial Plants by Selecting Maladapted Pathogen Strains
Source: Evol Appl. 2025 Jul 9;18(7):e70123. doi: 10.1111/eva.70123 (PMC12241718; doi:10.1111/eva.70123)
Supplement: Supplementary file 1 — Figure S1. Percentage of living hosts over the years since the pathogen introduction. Figure S2. Percentage of infected hosts over years, since the pathogen introduction. Figure S3. Epidemic development following pathogen introduction. Figure S4. Percentage of healthy compartments over time since pathogen introduction. Figure S5. Percentage of healthy compartements, at equilibrium, as a function of the percentage of resistant hosts in the landscape. Figure S6. Percentage of healthy compartments, at equilibrium, as a function of the percentage of resistant hosts in the landscape, in additionnal scenarios. Figure S7. Percentage of healthy compartments, at equilibrium, as a function of the percentage of resistant hosts in the landscape, in additional scenarios involving a saprotrophic pathogen. Figure S8. Percentage of healthy compartments, at equilibrium, as a function of the percentage of resistant hosts in the landscape, in additional scenarios. Figure S9. Proportion of healthy compartments over the years since pathogen introduction. Figure S10. Number of resistant healthy compartments over time following pathogen introduction. Figure S11. Number of susceptible healthy compartments over time following pathogen introduction. Figure S12. Mean infection strategy value of pathogen populations established on susceptible (left column) and resistant hosts (right column), over years following pathogen introduction. Figure S13. Mean variance (Vw) of infection strategy values within pathogen populations established on susceptible and resistant hosts. Figure S14. Genetic differentiation (QST) for infection strategy at year 20, between the population of pathogen established on susceptible hosts and the one established on resistant hosts. Figure S15. Variance (Vb) of mean infection strategy values of the pathogen populations established on susceptible and resistant hosts. [file EVA-18-e70123-s001.pdf]

Supplementary figures

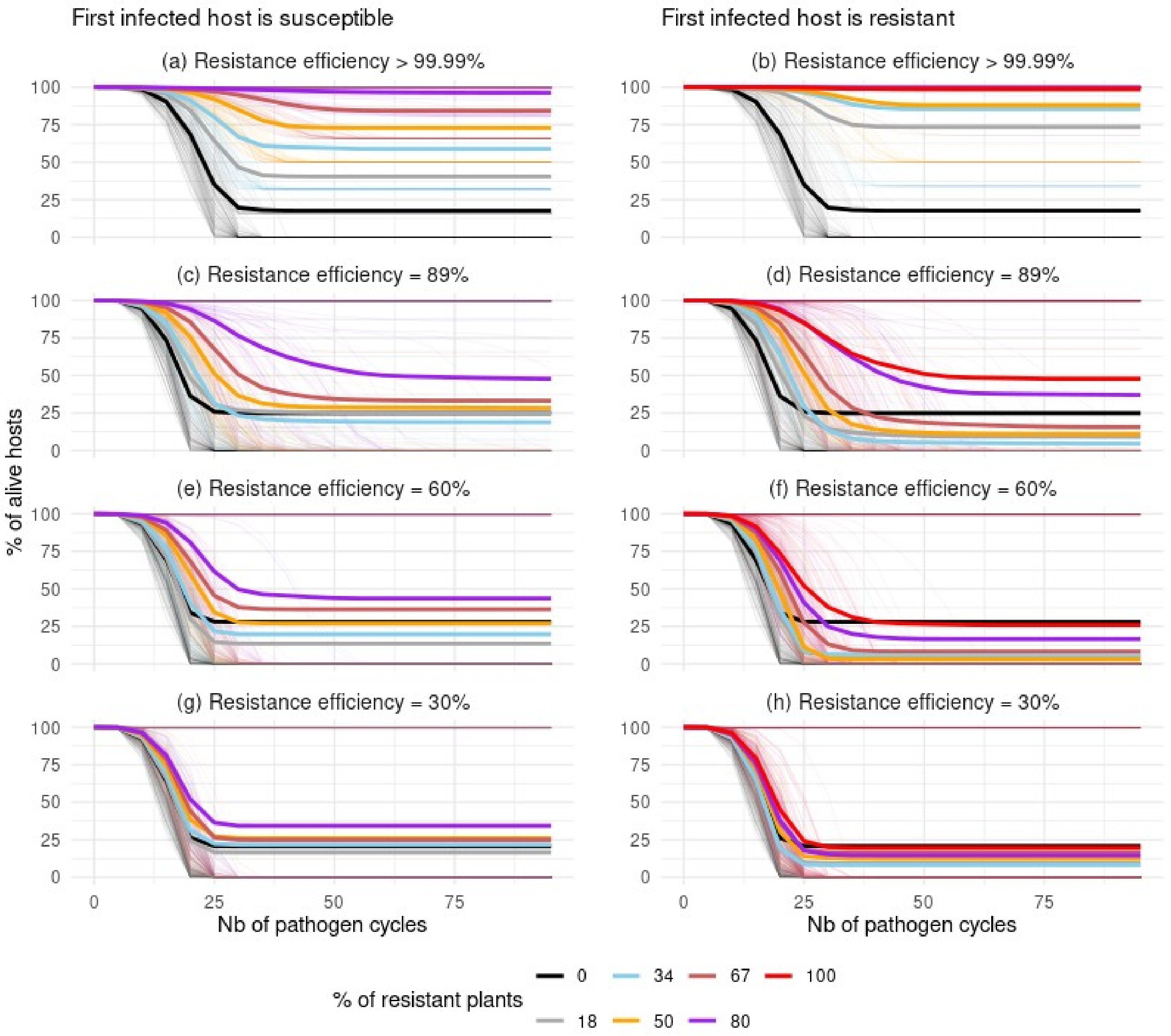

Figure S1. Percentage of living hosts over the years since the pathogen introduction. The first infected host was either susceptible (left column), or resistant (right column). In these scenarios, dead host plants were left unreplaced. Each light line corresponds to the values observed within a single replicate. Each thick line represents the mean of 96 replicates of a scenario.

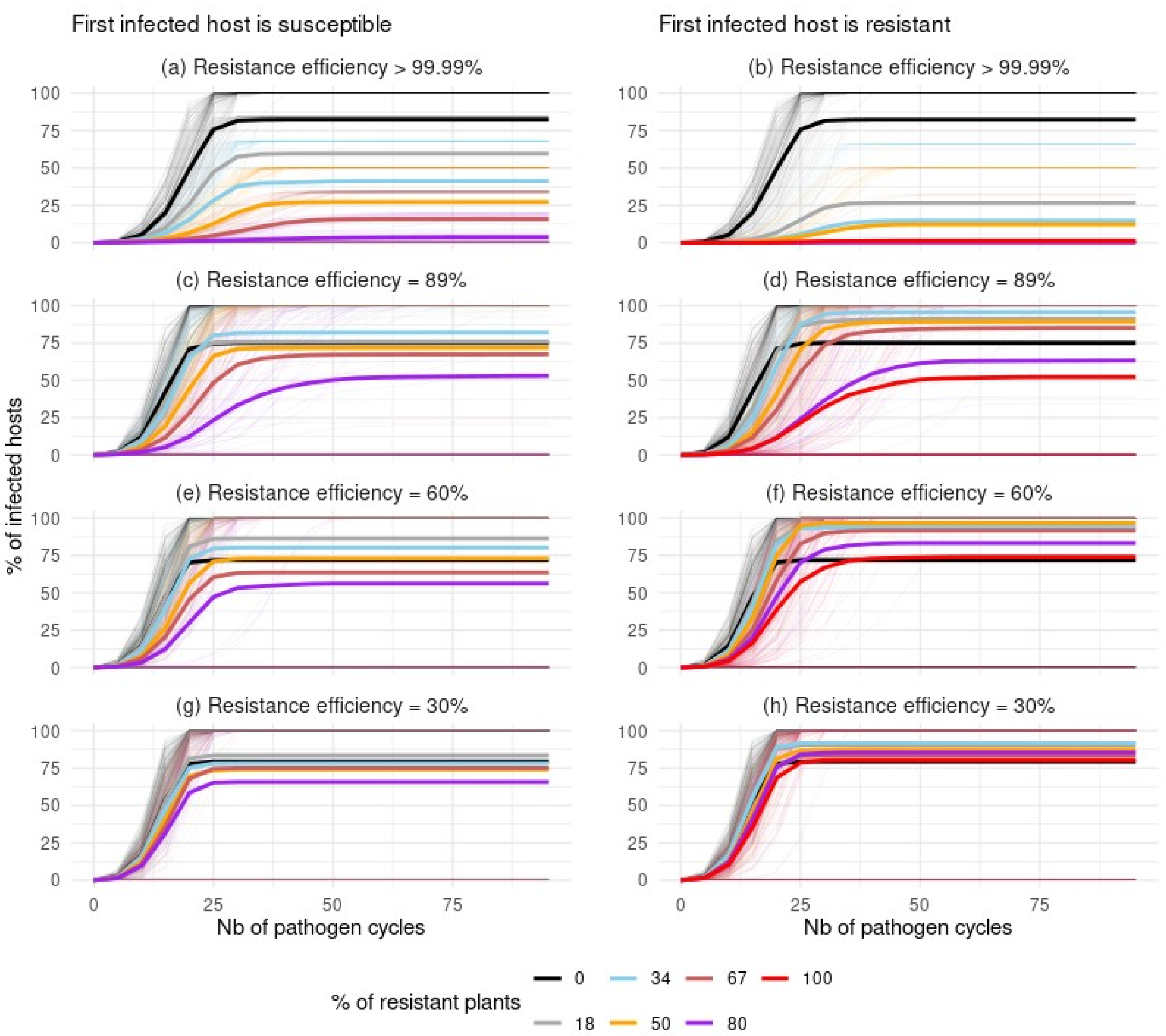

Figure S2. Percentage of infected hosts over years, since the pathogen introduction. The first infected host was either susceptible (left column), or resistant (right column). In these scenarios, dead host plants were left unreplaced. Each light line corresponds to the values observed within a single replicate. Each thick line represents the mean of 96 replicates of a scenario.

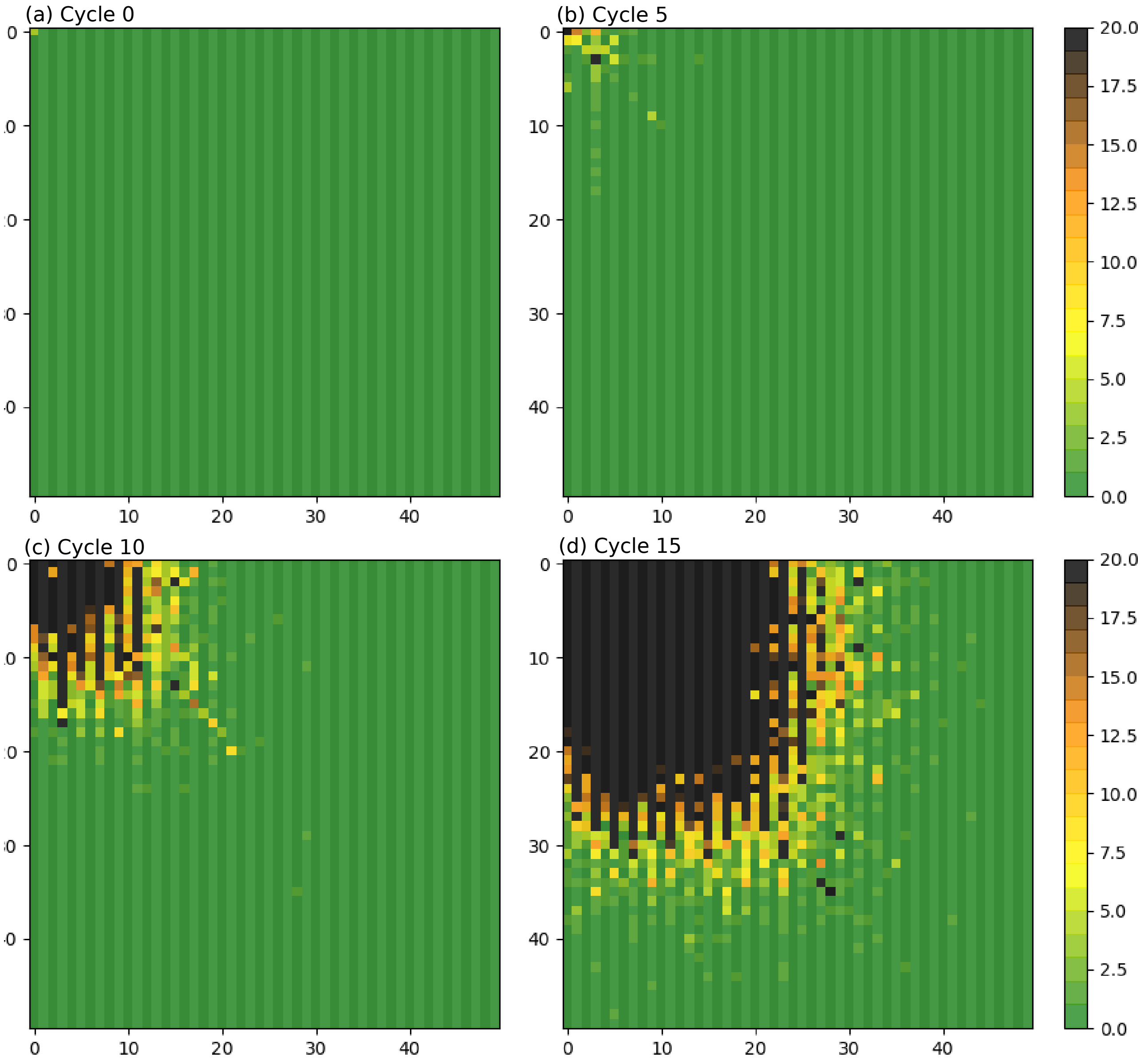

Figure S3. Epidemic development following pathogen introduction. Here the resistance used was of moderate efficiency, initially reducing the probability of pathogen establishment by 89%. Fifty per cent of resistant hosts were deployed in evenly spaced rows (dark lines). Four compartments of a resistant host were initially infected by two genotypes. The initially infected host was located in the upper-left corner of the landscape (see the light green point, panel (a)). The colour of each plant represents the mean number of infections of the corresponding plant. The values represented here are the values obtained in one of the 96 replicates simulated for this scenario.

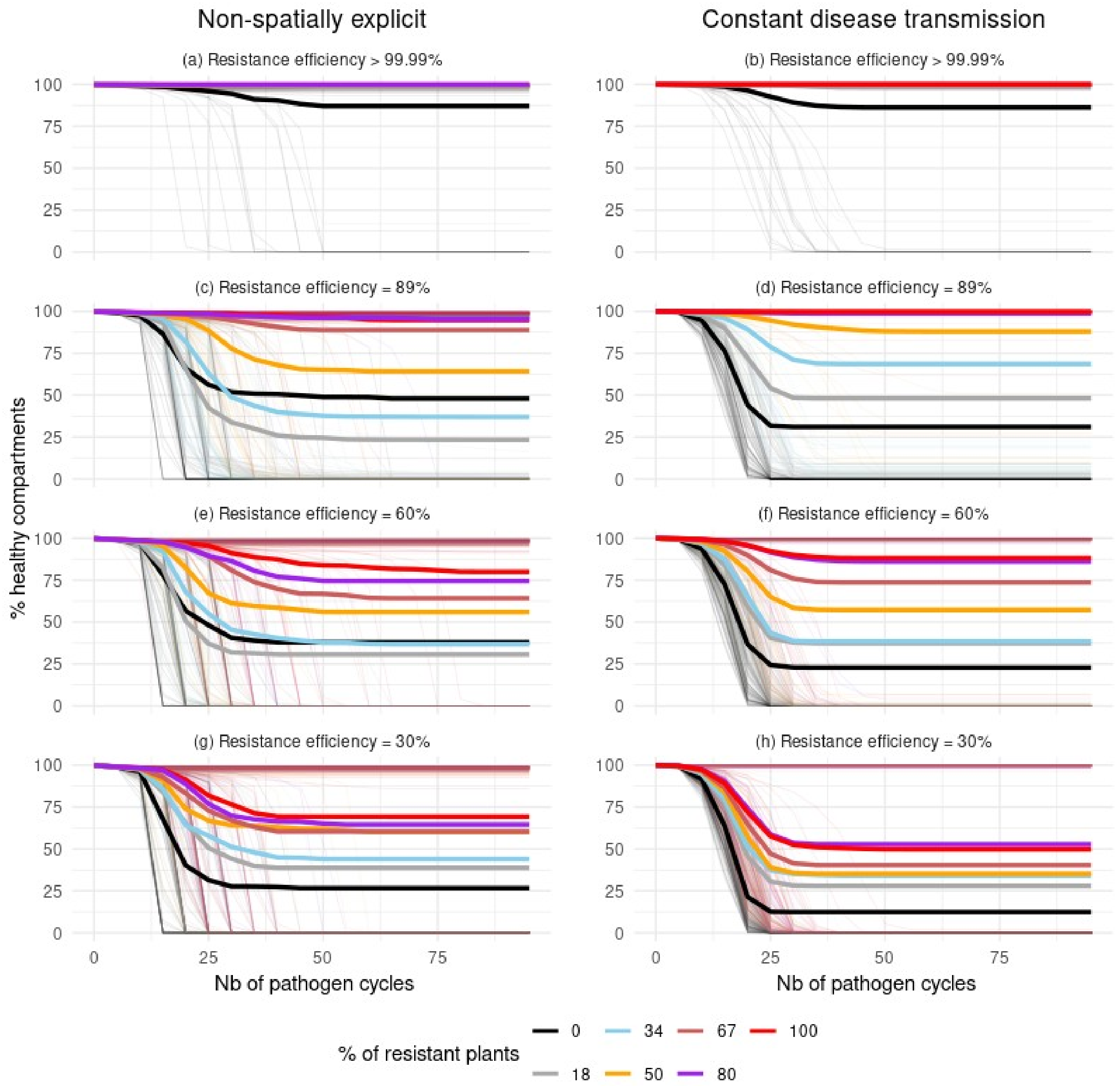

Figure S4. Percentage of healthy compartments over time since pathogen introduction. These predictions resulted from simplified versions of the model. Left column: non-spatially explicit version of the model. Right column: within- and between-plant pathogen propagation were not mechanistically linked. In these scenarios, dead host plants were left unreplaced. Each light line corresponds to the values observed within a single replicate. Each thick line represents the mean of 96 replicates of a scenario.

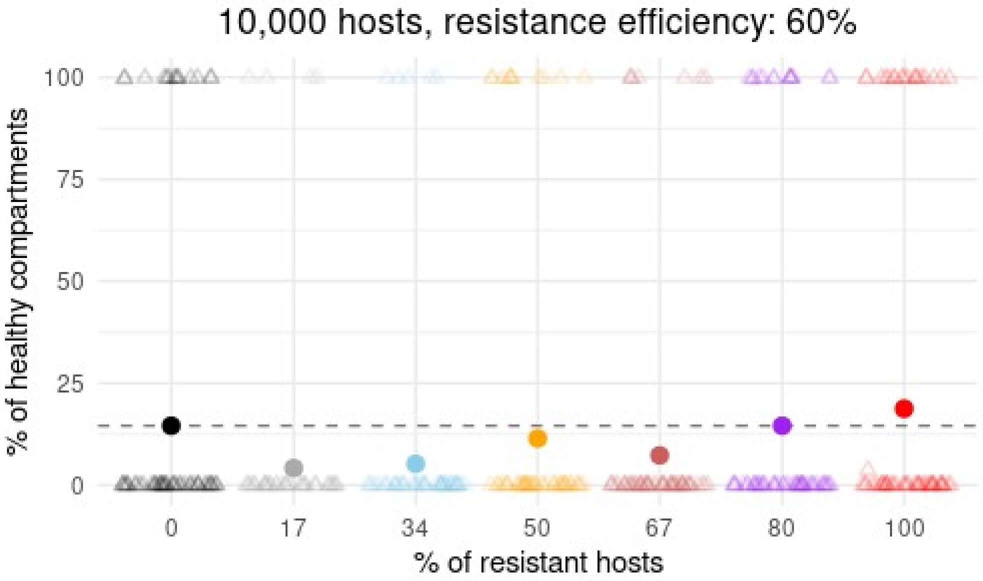

Figure S5. Percentage of healthy compartements, at equilibrium, as a function of the percentage of resistant hosts in the landscape. In these scenarios, the host landscapes comprised 10,000 plants and the efficiency of the resistance used was moderate. The first infected host was here resistant. Each point represents the mean of 96 replicates of a scenario. Each light triangle indicates the percentage of infected hosts within a single replicate.

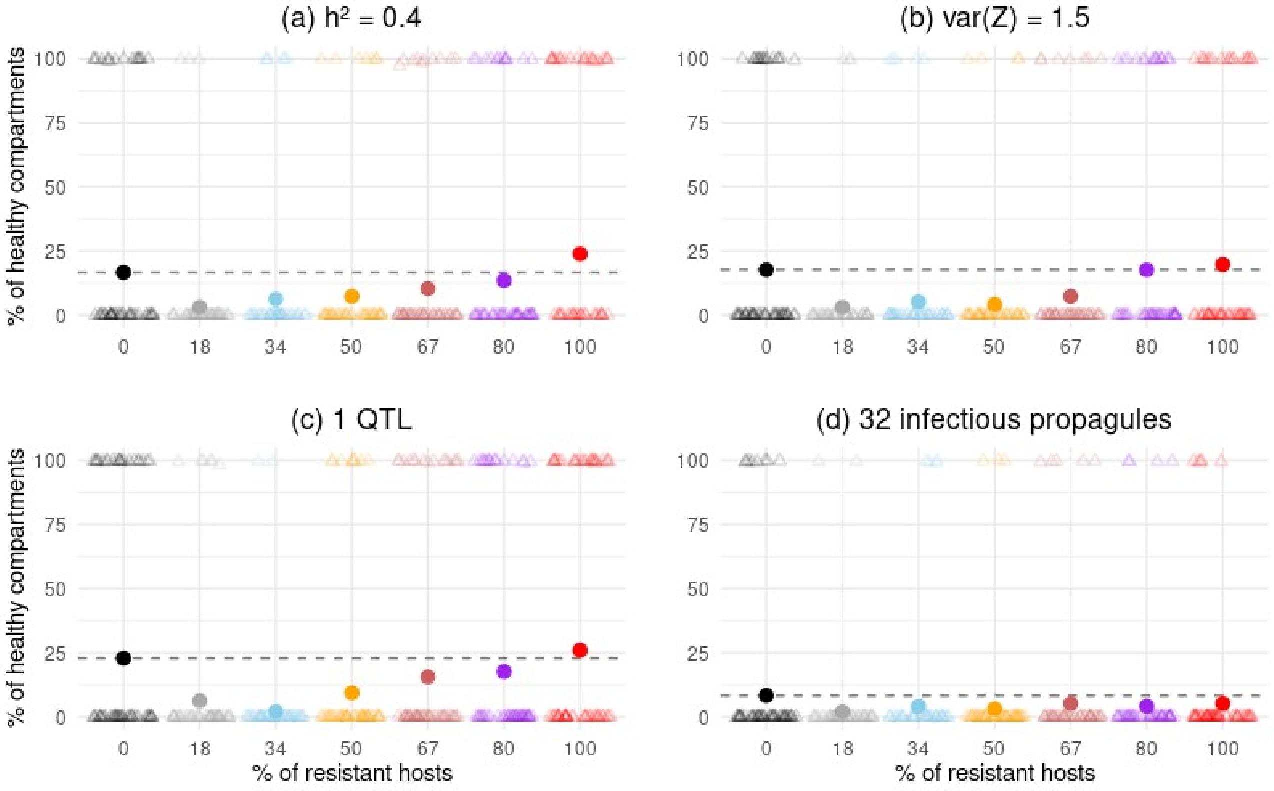

Figure S6. Percentage of healthy compartments, at equilibrium, as a function of the percentage of resistant hosts in the landscape, in additionnal scenarios. The resistance simulated was of moderate efficiency, the first infected plant was resistant, and dead plants were not replaced. h^2: heritability of the infection strategy, *var(Z)*: variance of the infection strategy. Each light triangle represents the percentage of alive hosts within one single replicate simulated. Each point is the mean percentage across 96 replicates for a given scenario.

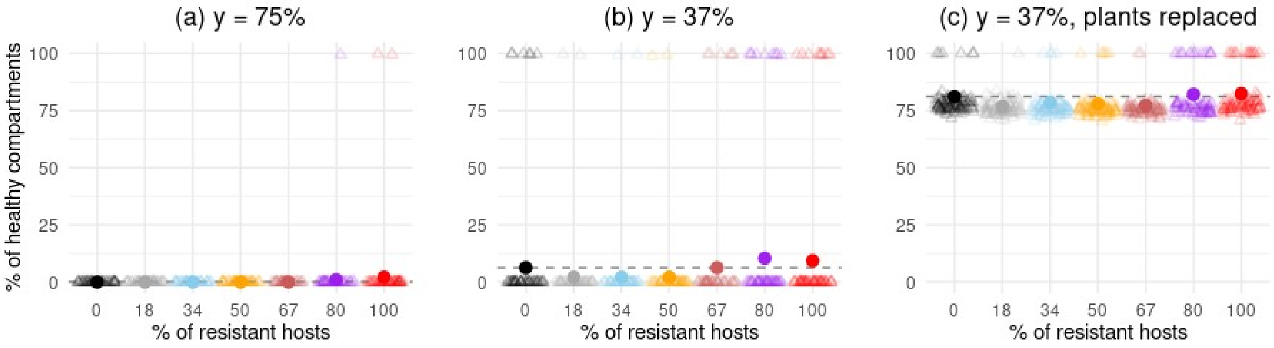

Figure S7. Percentage of healthy compartments, at equilibrium, as a function of the percentage of resistant hosts in the landscape, in additional scenarios involving a saprotrophic pathogen. Panels (a) and (b): dead hosts were not replaced by healthy ones. Panel (c): dead hosts were replaced. *y* indicates the probability that a fungal genotype survived the death of its host. The resistance simulated was of moderate efficiency, and the first infected plant was resistant. Each light triangle represents the percentage of alive hosts within one single replicate simulated. Each point is the mean percentage across 96 replicates for a given scenario.

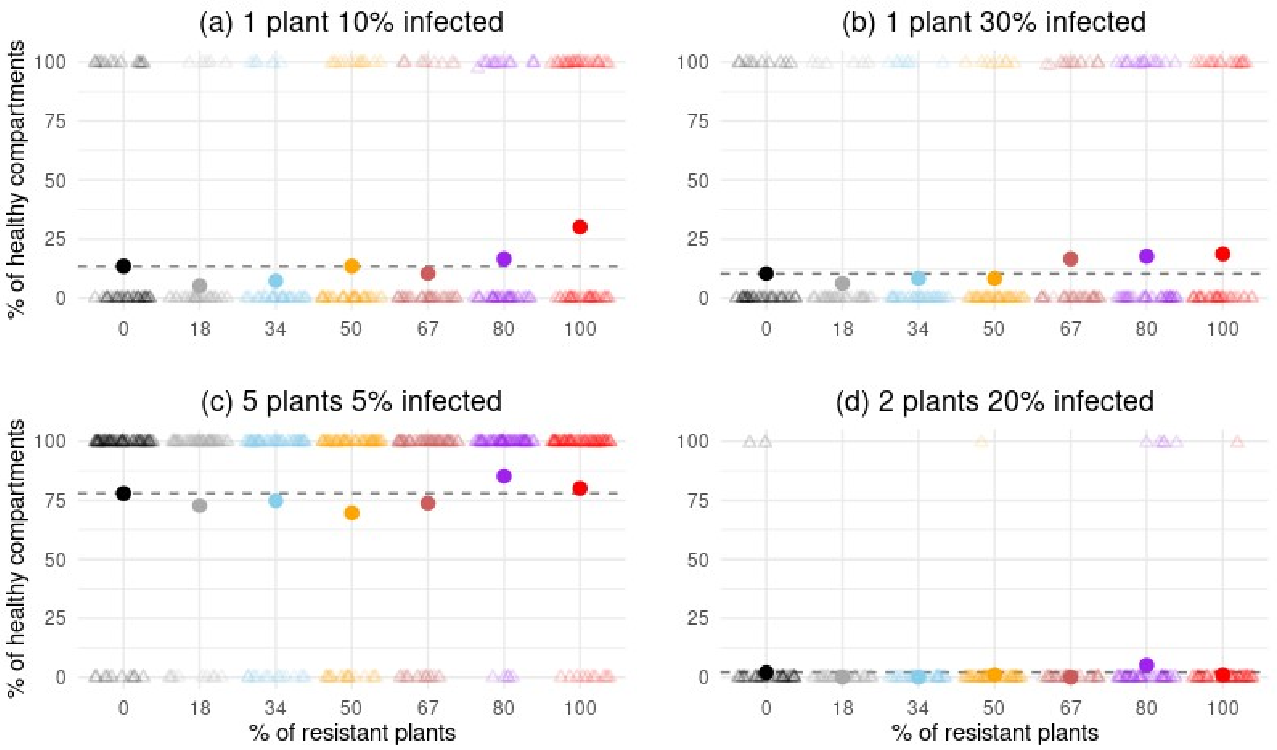

Figure S8. Percentage of healthy compartments, at equilibrium, as a function of the percentage of resistant hosts in the landscape, in additional scenarios. In these scenarios, both the number of hosts initially infected and their initial pathogen load were varied. The resistance simulated was of moderate efficiency, the first infected plant was resistant and there was no replacement of dead plants. Each light triangle represents the percentage of alive hosts within one single replicate simulated. Each point is the mean percentage across 96 replicates for a given scenario.

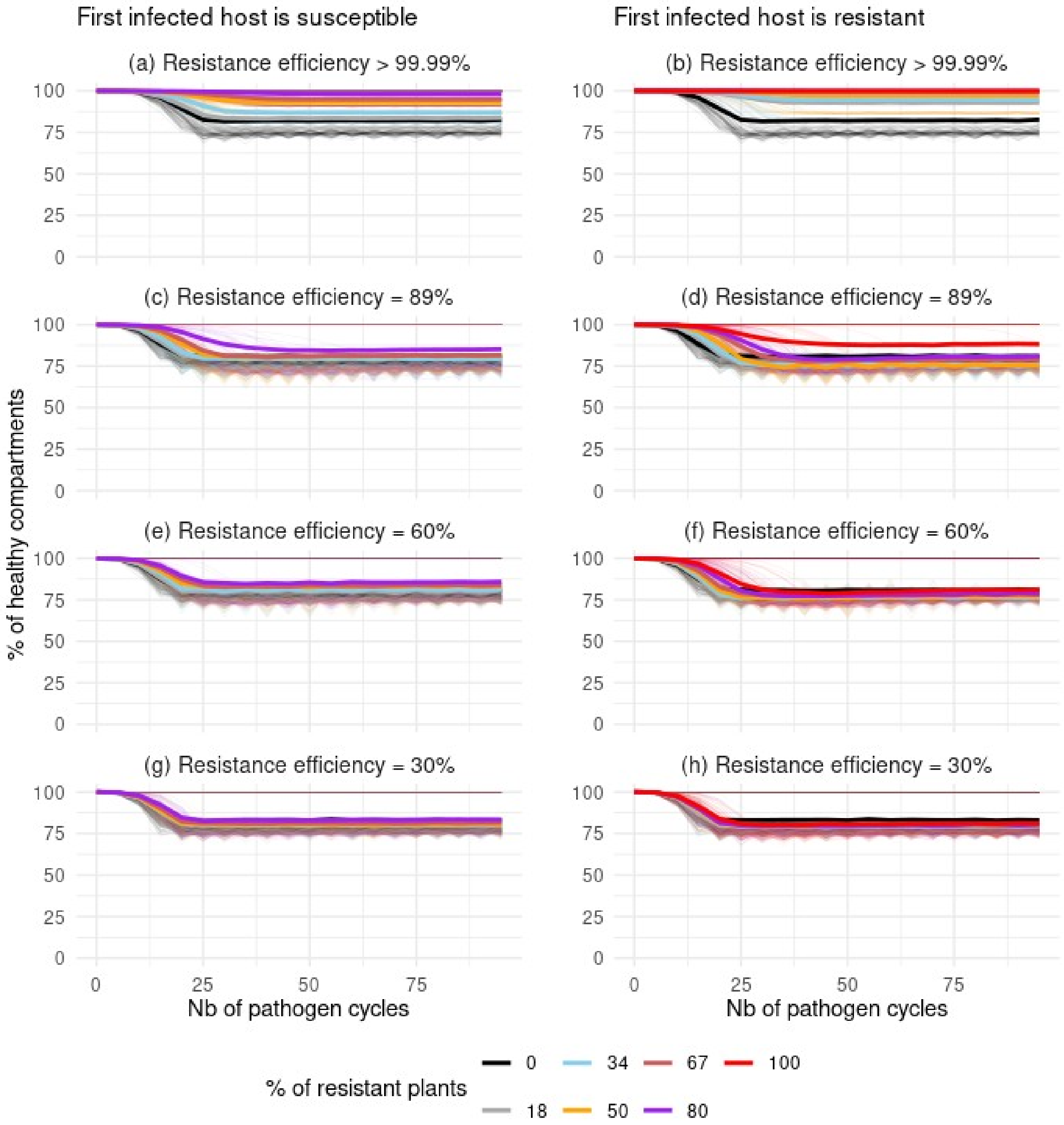

Figure S9. Proportion of healthy compartments over the years since pathogen introduction. Here dead plants were systematically replaced by entirely healthy plants. The first host infected was either susceptible (left column) or resistant (right column). Each light line represents the observations from a single replicate. Each thick line denotes the mean of 96 replicates of a scenario.

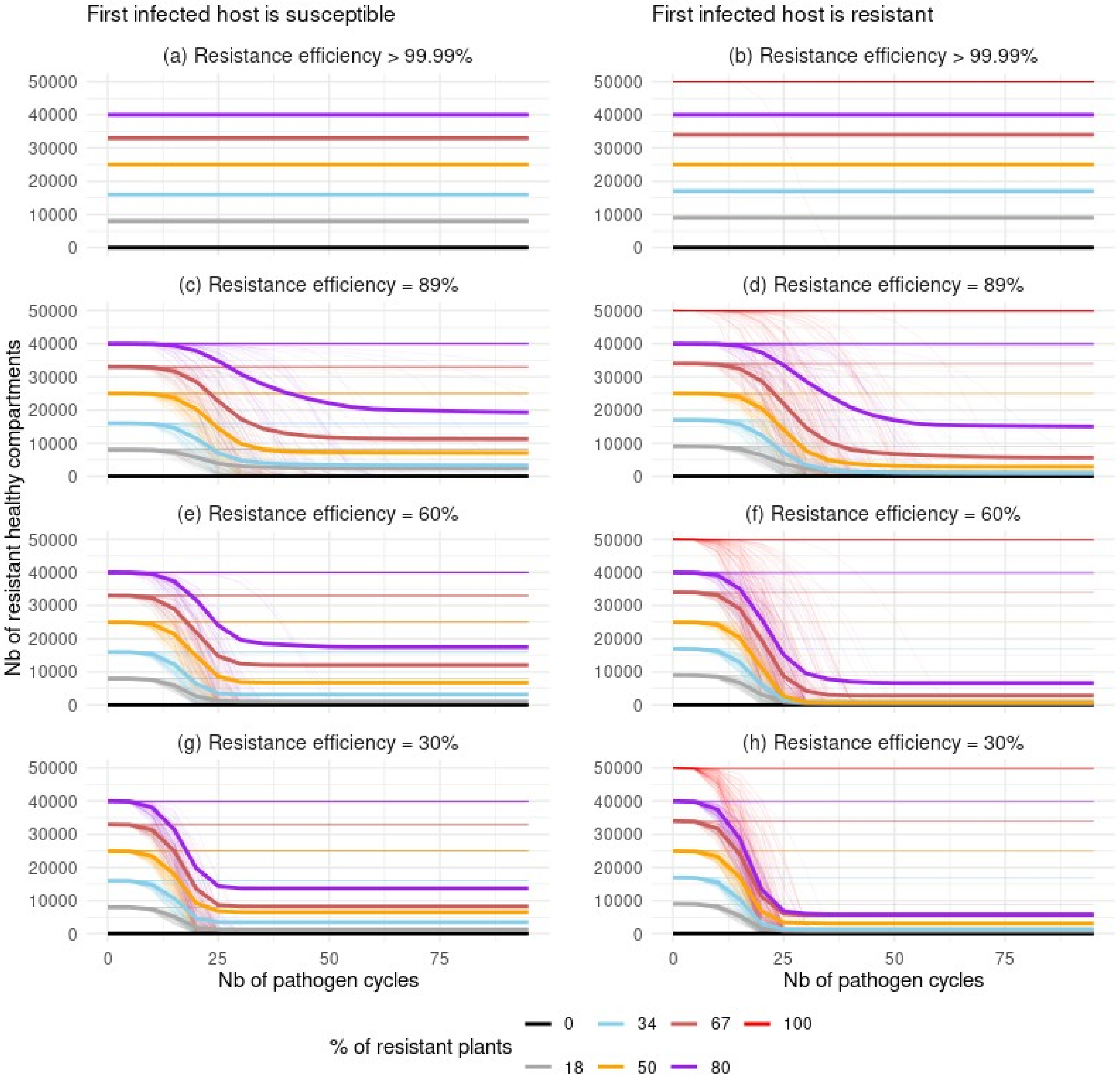

Figure S10. Number of resistant healthy compartments over time following pathogen introduction. The initial infected host was either susceptible (left column) or resistant (right column). Thin lines represent observations from each simulation replicate, while thick lines indicate the mean across 96 replicates for each scenario.

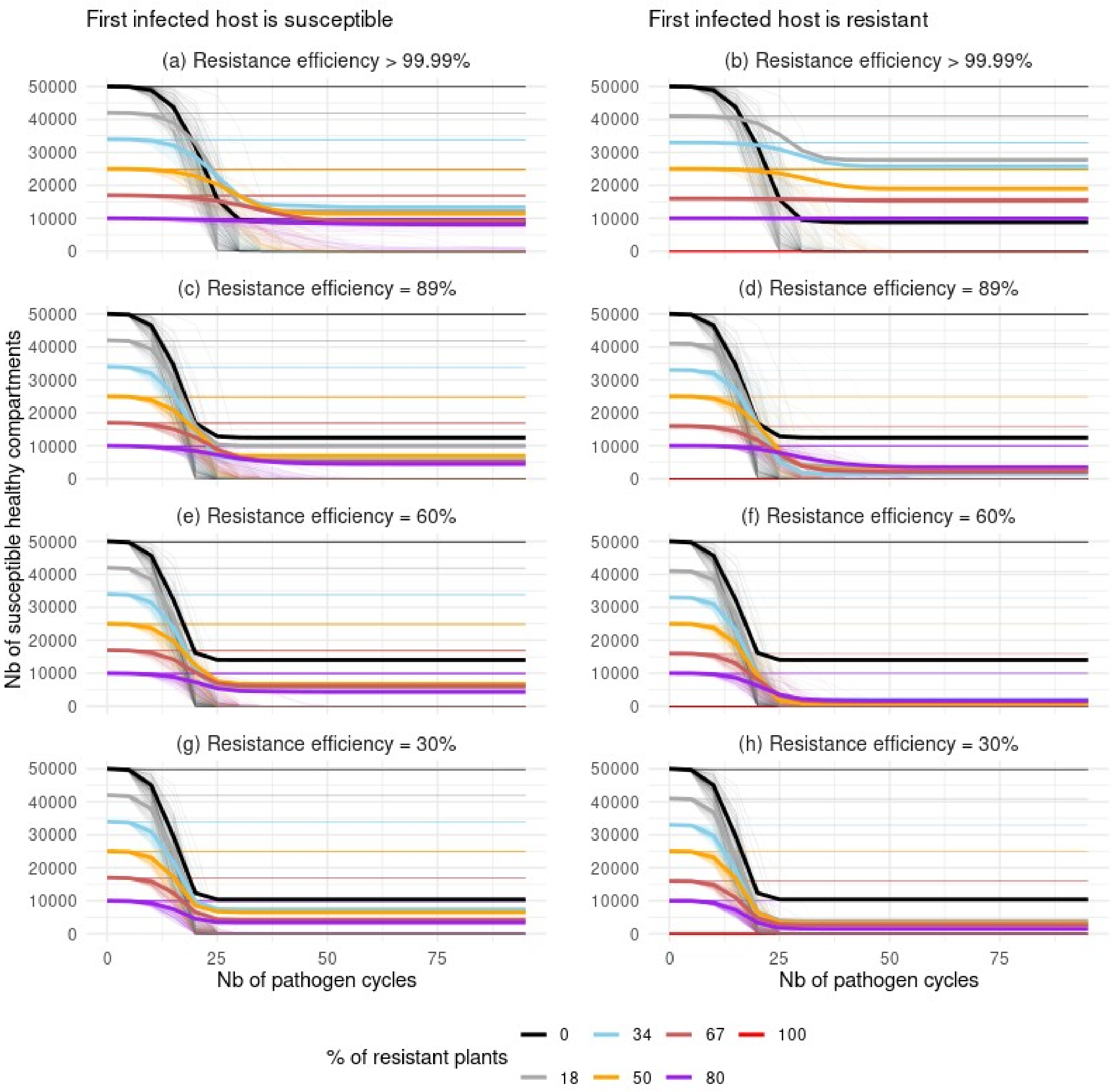

Figure S11. Number of susceptible healthy compartments over time following pathogen introduction. The initial infected host was either susceptible (left column) or resistant (right column). Thin lines represent observations from each simulation replicate, while thick lines indicate the mean across 96 replicates for each scenario.

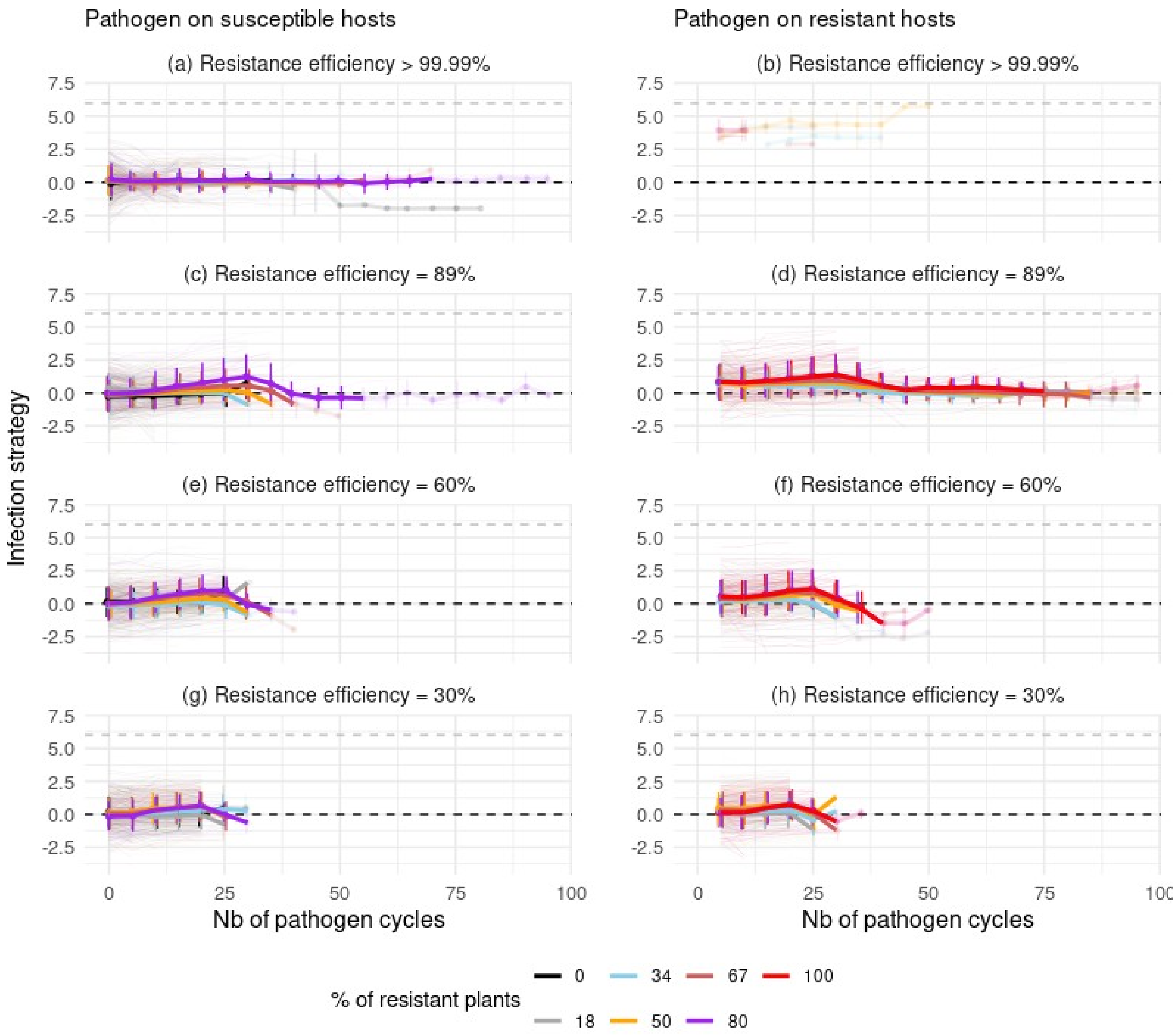

Figure S12. Mean infection strategy value of pathogen populations established on susceptible (left column) and resistant hosts (right column), over years following pathogen introduction. Here the first infected host was susceptible and there was no replacement of dead plants. The points connected by thick lines are the mean trait values computed over 96 replicates of a scenario. Each bar indicates the standard deviation of the corresponding mean value. Each light line represents the infection strategy within a single replicate. The opacity of the thick lines and bars was reduced when the number of non-null replicates fell below 10. The black and grey dashed lines correspond to the optimal infection strategy values that confer the highest chance of infecting susceptible and resistant hosts, respectively.

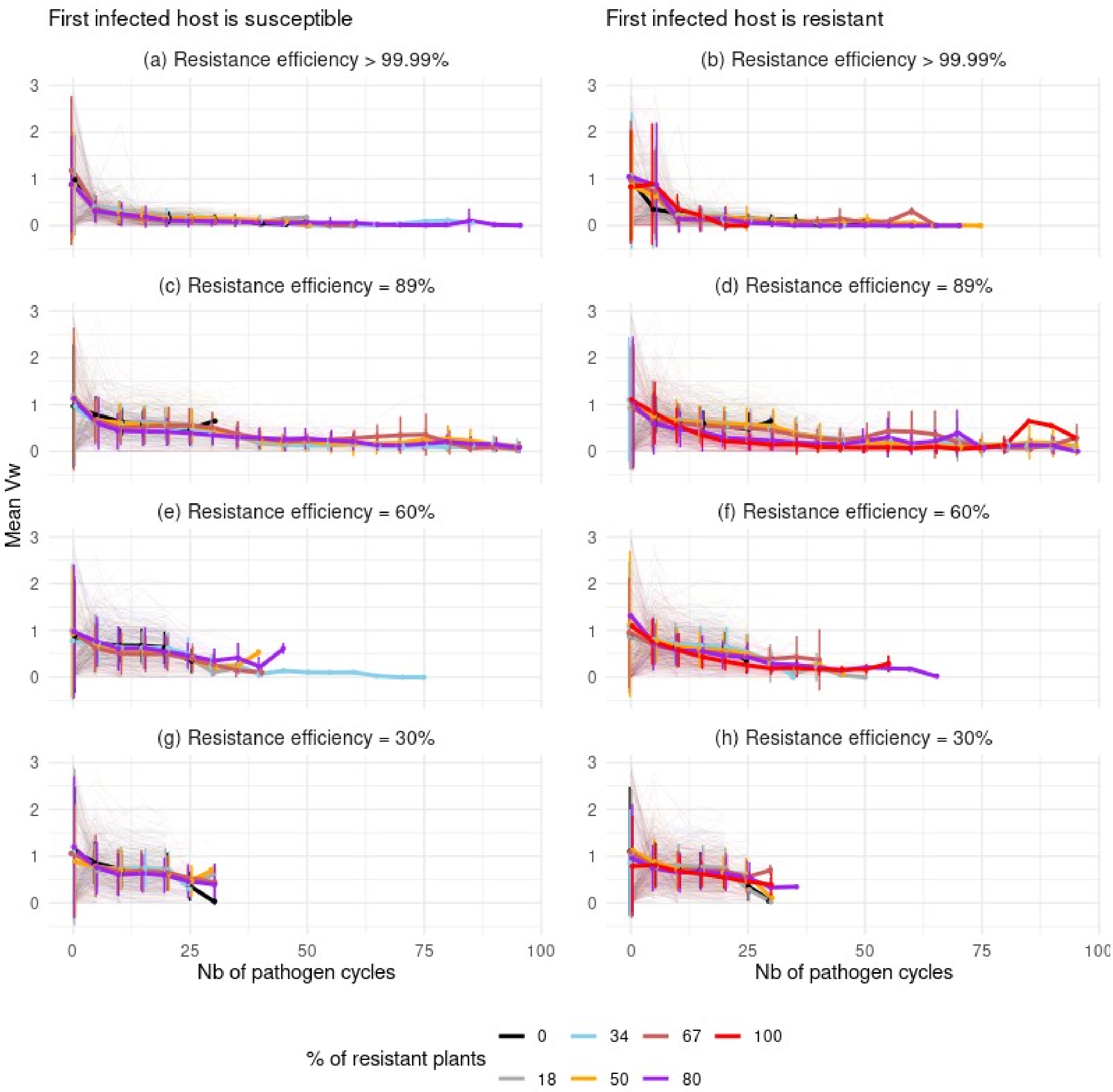

Figure S13. Mean variance (Vw) of infection strategy values within pathogen populations established on susceptible and resistant hosts. The first infected host was either susceptible (left column) or resistant (right column). The thick lines represent the mean infection strategy values calculated across 96 replicates of a scenario. Each bar indicates the standard deviation of the corresponding mean value. Each light line represents the mean variance within a single replicate.

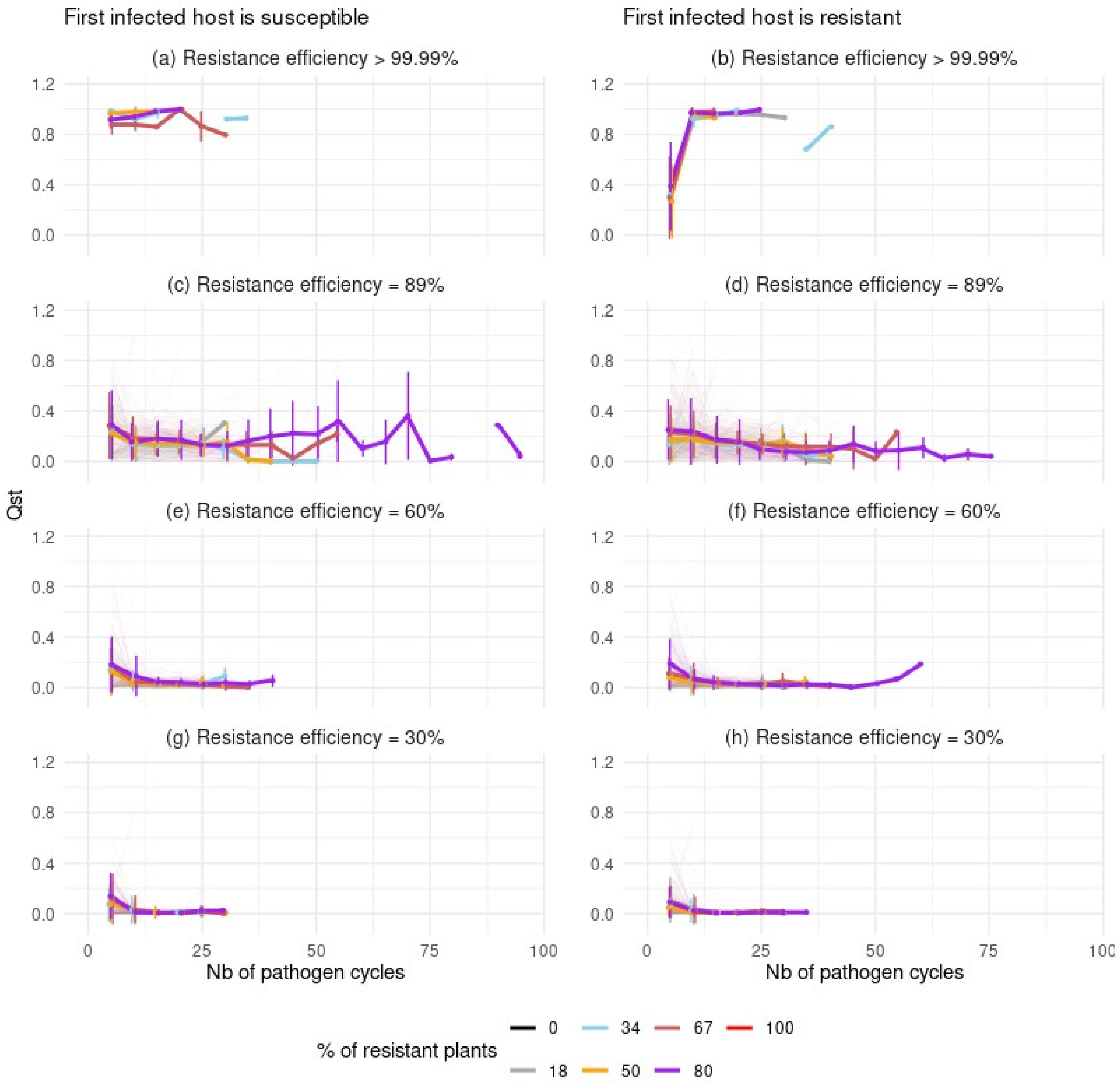

Figure S14. Genetic differentiation (Q_{ST}) for infection strategy at year 20, between the population of pathogen established on susceptible hosts and the one established on resistant hosts. The first infected host was either susceptible (left column), or resistant (right column). The thick lines represent the mean infection strategy values calculated across 96 replicates of a scenario. Each bar indicates the standard deviation of the corresponding mean value. Each light line represents the mean variance within a single replicate.

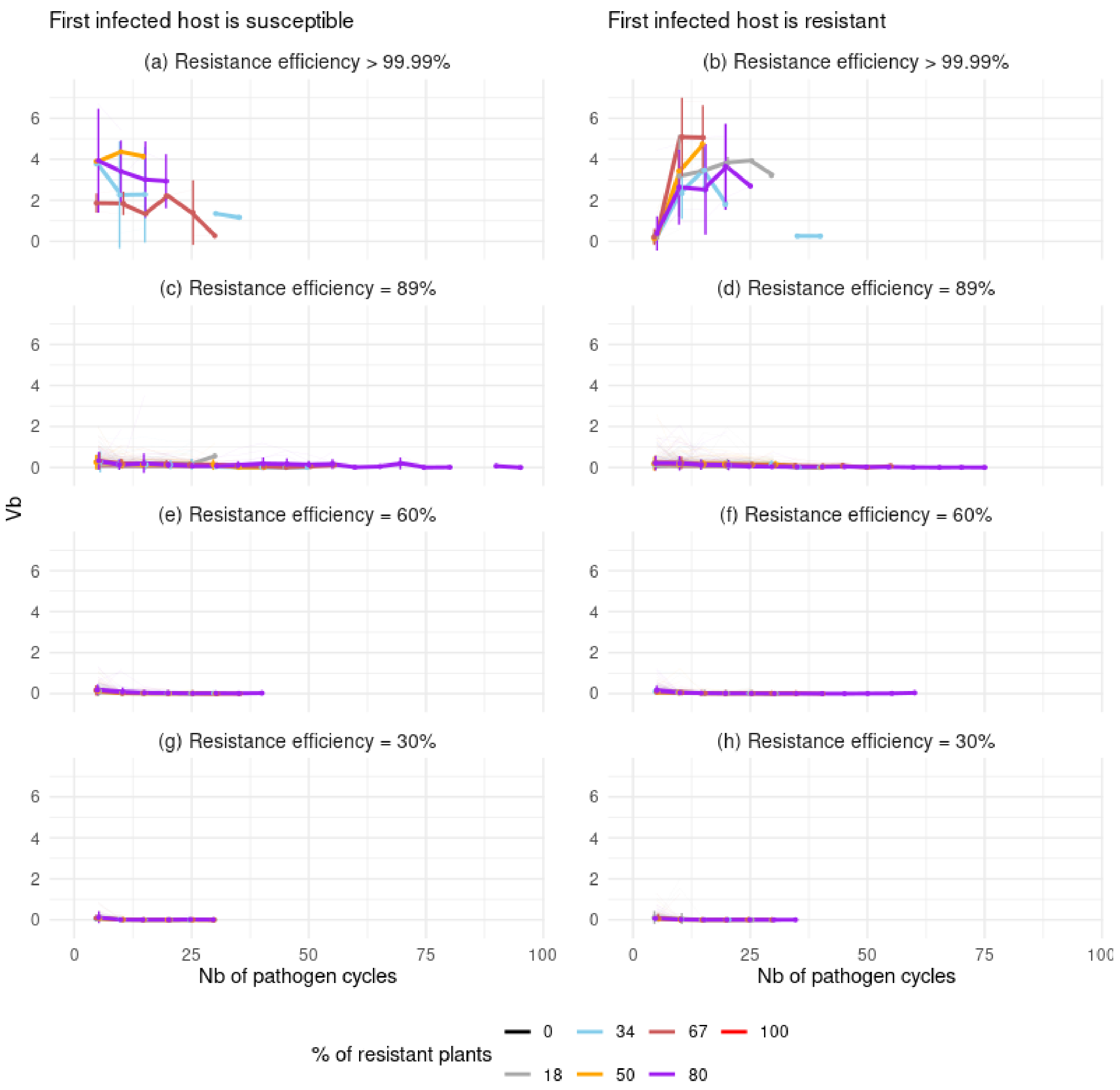

Figure S15. Variance (*Vb*) of mean infection strategy values of the pathogen populations established on susceptible and resistant hosts. The first infected host was either susceptible (left column), or resistant (right column). The thick lines represent the mean infection strategy values calculated across 96 replicates of a scenario. Each bar indicates the standard deviation of the corresponding mean value. Each light line represents the mean variance within a single replicate.
